# Synapses do not facilitate prion-like transfer of alpha-synuclein: a quantitative study in reconstructed unidirectional neural networks

**DOI:** 10.1007/s00018-023-04915-4

**Published:** 2023-09-09

**Authors:** Josquin Courte, Ngoc Anh Le, Teng Pan, Luc Bousset, Ronald Melki, Catherine Villard, Jean-Michel Peyrin

**Affiliations:** 1 Faculté des Sciences et Technologie, Institut de Biologie Paris Seine, Sorbonne Universités, CNRS UMR 8246, INSERM U1130, Neurosciences Paris Seine, 75005 Paris, France; 2 Institut Curie, CNRS UMR 168, Université PSL, Sorbonne Universités, 75005 Paris, France; 3 Institut François Jacob, (MIRCen), CEA and Laboratory of Neurodegenerative Diseases, CNRS, 92260 Fontenay-Aux-Roses, France

**Keywords:** Microfluidics, Neuronal culture system, Neural networks reconstruction, Axon growth, Brain-on-a-chip, Organ-on-a-chip, Alpha-synuclein, Prion-like, Parkinson’s disease, Synucleinopathies

## Abstract

Alpha-synuclein (aSyn) aggregation spreads between cells and underlies the progression of neuronal lesions in the brain of patients with synucleinopathies such as Parkinson’s diseases. The mechanisms of cell-to-cell propagation of aggregates, which dictate how aggregation progresses at the network level, remain poorly understood. Notably, while prion and prion-like spreading is often simplistically envisioned as a “domino-like” spreading scenario where connected neurons sequentially propagate protein aggregation to each other, the reality is likely to be more nuanced. Here, we demonstrate that the spreading of preformed aSyn aggregates is a limited process that occurs through molecular sieving of large aSyn seeds. We further show that this process is not facilitated by synaptic connections. This was achieved through the development and characterization of a new microfluidic platform that allows reconstruction of binary fully oriented neuronal networks in vitro with no unwanted backward connections, and through the careful quantification of fluorescent aSyn aggregates spreading between neurons. While this allowed us for the first time to extract quantitative data of protein seeds dissemination along neural pathways, our data suggest that prion-like dissemination of proteinopathic seeding aggregates occurs very progressively and leads to highly compartmentalized pattern of protein seeding in neural networks.

**Supplementary Information:**

The online version contains supplementary material available at 10.1007/s00018-023-04915-4.

## Background

Chronic neurodegenerative diseases (ND) such as synucleinopathies are characterized by slow and progressive neuronal dysfunctions that occur over years in patients. It is now well established that a subset of neurodegenerative syndromes involves the progressive accumulation of abnormal protein aggregates in neurons [1]. Alpha-synuclein (aSyn) aggregation, which is associated with the formation of Lewy Bodies (LB), is one of the major hallmarks of synucleinopathies and is hypothesized to play an important role in the etiology of Parkinson’s disease (PD)-related neuronal dysfunctions [2]. Recent evidence from neuropathological studies shows that LB accumulation progresses both spatially and temporally in the brains of affected patients and follows a rostro-caudal stereotyped pattern [3]. Somewhat akin to the pathogenic form of the prion protein, aggregated aSyn spreads from cell to cell and amplifies by seeding the aggregation of its soluble counterpart [4–8]. Protein aggregates build up as structural polymorphic structures holding specific biological activities reminiscent of prion strains [9–13]. Consequently, the “prion-like hypothesis” posits that the propagation of aSyn aggregation in brain networks underlies the stereotyped development of neuronal lesions. Inter-neuronal propagation of aggregation can be divided in three major steps: (1) uptake of exogenous aggregates or generation of endogenous aggregates by first-order neurons, (2) transfer of exogenous and/or neo-generated aggregates to second-order neurons and (3) seeding of aggregation in second-order neurons. Yet both the molecular and cellular mechanisms involved in the spatial spreading of aSyn aggregates between neurons and along neural pathways remain poorly understood. The prion-like aggregation of aSyn gives rise to several different sized aggregates with structurally distinct characteristics, from large amyloid fibrils to smaller non-amyloid aggregates. It has been proposed that while larger aggregates are able to seed the aggregation of the endogenous aSyn proteins, smaller oligomers are more toxic [14]. Different species of aggregates which mimic naturally occurring assemblies have been generated in vitro from recombinant aSyn and have been shown to exhibit distinct biological and structural properties, such as large Fibrils able to seed the aggregation of endogenous aSyn and less structured Oligomers which do not seed aggregation [15, 16]. Several studies suggest that aSyn aggregates can be conveyed in extracellular medium and spread to neighboring cells naked, or through small extracellular vesicles or tunneling nanotubes in immortalized cell lines and primary neuronal cultures [17–21]. The respective contribution of these mechanisms to aSyn aggregates transfer remains unclear. Because sequentially affected brain regions are synaptically connected, synapses are postulated to play a role in aSyn aggregates trans-neuronal spreading [22, 23]. Indeed, synapses tightly link neuronal membranes and harbor putative receptors of aSyn aggregates [24]. Moreover, synapses are membrane remodeling hotspots [25] and contain most of the pool of soluble -and vulnerable to seeding- aSyn in their presynaptic moiety. Yet, their role in aSyn transfer has not been clearly evaluated, and synaptic connectivity alone does not predict the pattern of aggregation spreading in the brain. Indeed, brain regions which share a lot of synaptic connections with brain nuclei developing aggregates early in the disease, such as the locus coeruleus, are affected later than some more sparsely connected regions [26–28]. The role of synaptic structures in facilitating inter-neuronal transfer of aSyn aggregates thus remains uncertain. It also remains unclear how neuronal identity and aSyn expression affect inter-neuronal transfer of aSyn aggregates.

These questions are difficult to address in vivo. We propose that in vitro models permitting the manipulation of neural networks may allow to quantitatively evaluate how aSyn aggregates structure, as well as neuronal identity and synaptic connectivity might affect the inter-neuronal propagation of aSyn aggregation [19, 29, 30]. Here, we describe a new microfluidic design which allows for the first time the full control of axonal outgrowth direction between two fluidically isolated culture compartments, therefore permitting the reconstruction of fully oriented binary neuronal networks. Using that platform, we demonstrate that while seeding aggregates (Fibrils) spread anterogradely from first-order to second-order neurons through axons, this process is limited and progressive with only ~ 1/100 of the inoculated Fibrils diffusing to second-order neurons over a three days period. Inter-neuronal transfer of small non-seeding aggregates (Oligomers) is one order of magnitude more efficient. An increase in maturity and therefore synaptic connectivity of the inoculated networks did not lead to increased transfer of either aggregates, but rather tended to diminish the transfer rate. Neuronal identity of the presynaptic neurons did not impact the anterograde transfer rate of Fibrils. Seeding of aSyn aggregation in the post-synaptic neurons 10 days post pre-synaptic inoculation of the network was weak and started in axonal projections. Altogether, our observations suggest that seeding aggregates may be sieved by intact synapses, leading to a delayed and compartmentalized propagation of aSyn aggregation in neural networks.

## Results

### Reconstruction of fully oriented neural networks in vitro

Acknowledging that protein aggregates such as aSyn Fibrils are actively transported from terminal endings toward neuronal soma [31], studying trans-neuronal transfer of protein aggregates in artificially reconstructed neuronal networks composed of separately perfusable “emitting” and “receiving” neuronal populations requires that virtually no axons from receiving neurons shoots back toward emitting ones. While we previously described microfluidic platforms enforcing directional connectivity between A and B neurons [32, 33] those systems did not allow to obtain fully oriented networks, as a small proportion of axons from neurons in the receiving compartment could send their axons back toward the emitting chamber. Preliminary experiments conducted with those classical “axonal diode” microchips showed that fluorescent aSyn Fibrils may transfer from one chamber to the other via retrograde transport through those few “backward” axon terminals (data not shown).

**Fig. 1:**
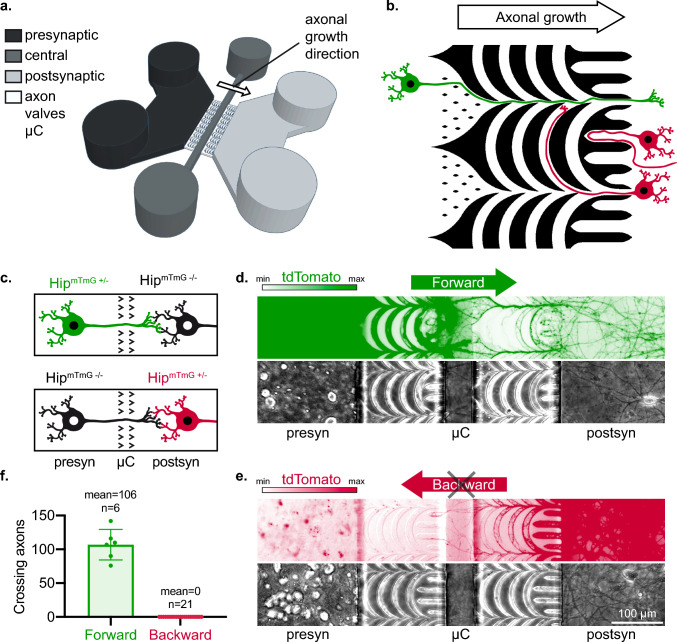
In vitro reconstruction of fully oriented neural networks. **a** Schematic representation of the hollow parts of the microfluidic culture device for oriented neural networks reconstruction. Three main compartments (black: presynaptic compartment; dark gray: central compartment; gray: postsynaptic compartment) are accessible though fluidic inlets/outlets and linked through structured axon valves microchannels (µC) (white). The presynaptic and postsynaptic compartments are seeded with neurons while the central compartment improves the fluidic isolation between the culture compartments and permits the examination of isolated axonal tracts. The white arrow indicates the imposed direction of axonal growth in the microchannels. The height of culture chambers is 50 µm while microchannels are 3 µm high. **b** Principle of unidirectional axonal filtration in a single microchannels array (2 sequential arrays are present in the microfluidic culture system). In green, neurons originating from the presynaptic chamber can cross the microchannels array while in red, neurons originating from the postsynaptic chamber are either filtered by axonal traps or by arches microchannels. **c** Schematic representation of the experimental design. Hip^mTmG+/–^ > Hip^mTmG−/−^ (tdTomato+  > tdTomato-) networks permitted to evaluate forward axonal growth (in green) while Hip^mTmG−/−^ > Hip^mTmG+/–^ permitted to observe backward axonal growth (in red). The “ > ” signs denote the forward bias in axonal growth through the two arrays of axon valves. **d**, **e** Representative epifluorescence microscopy fields of chimeric reconstructed neural networks after 19 days of culture. For preserving a uniform color code for the whole figure, a different look-up table was used for tdTomato signal in panels d and e, depending on if Hip^mTmG+/–^ cells were seeded in the presyn or postsyn compartment. **f** Quantification of forward and backward axonal outgrowth in chimeric Hip > Hip networks, with individual points representing individual culture devices. The number n of individual culture devices is indicated on the graph, from *N* = 3 individual experiments. Error bars show standard deviation

In order to tackle that drawback, we developed a new compartmentalized culture system which satisfies the requirements for studying trans-neuronal propagation of protein particles with prion-like properties by enforcing full (100%) unidirectional axonal growth and synaptic connectivity between emitting and receiving neurons. We built on our previous approaches for guiding the direction of axonal growth [29, 32, 33] to design new asymmetric microchannels which completely restrict axonal growth to a single direction. We took inspiration from the fluidic “Tesla” valves (US patent #US001329559) to name them “axon valves” (Fig. 1a and b, detailed dimensions of the microchannels can be found in Supp Fig. 1). In the “forward” direction, axons are funneled by large openings (~ 80 µm) into the microchannels maze which they can cross by following a “zig-zag” path (Fig. 1b, green neuron). In the “backward” direction, the initial size of microchannels openings is much smaller (6 µm), and axons which penetrate the maze are submitted to a series of divergent Y junctions (Fig. 1b, red neurons). As axons exhibit some degree of rigidity in their pathfinding behavior and tend to grow in the corners of rectangular microchannels, the path they are the most likely to follow sends them back to the compartment they came from, due to the presence of “arches-shaped” microchannels linking the divergent Y junctions [33]. In addition, “dead-end” microchannels trap some of the “backward” axons, with the goal of preventing them from growing alongside the walls until they find one of the narrow openings. We will call the axon emitting compartment “presynaptic” (presyn), central compartment “axonal” and receiving compartment “postsynaptic” (postsyn) from now on. We further biased axonal growth by seeding the postsyn compartment with ~ 4 times less neurons than the presyn compartment. It is noteworthy that, probably due to differences in trophic support, neuronal densities in the presyn compartment ended up 4- to 12-fold higher than in the postsyn culture compartment in later culture times (Supp Fig. 2b).

**Fig. 2:**
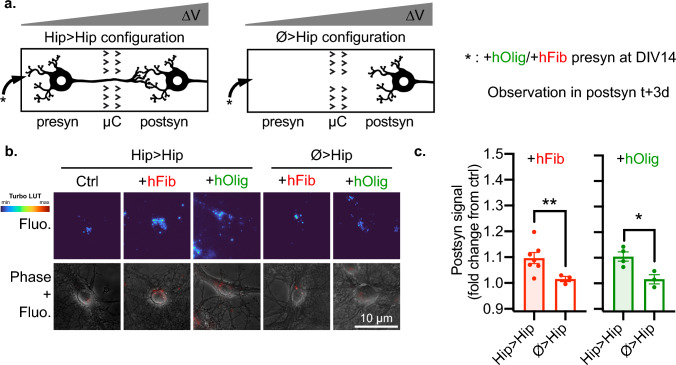
aSyn exogenous aggregates are actively transferred from neuron to neuron in an anterograde fashion. **a** Schematic representation of the experimental design. The presynaptic compartment of Hip>Hip or Ø>Hip networks was spiked with 500 nM hFib or 500 nM hOlig, and aggregates associated fluorescence was monitored in neuronal somas in the postsyn compartment 3 days later. **b** Representative epifluorescence microscopy fields of postsynaptic neurons. Top row, hFib associated fluorescence is shown with the Turbo heatmap (dark blue is zero, dark red is max, obtained from github.com/cleterrier/ChrisLUTs.). Bottom row, hFib fluorescence is in red and Phase signal in gray. **c** Quantification of the average fluorescence in the somas of postsynaptic neurons (fold change from signal in control condition: untreated Hip>Hip networks). hFib networks: *n* = 9–28, *N* = 4–7. hOlig networks: *n* = 10–16, *N* = 3–4. (n individual culture devices from N individual experiments). Unpaired t-test with Welch’s correction. **p < 0.01; *p < 0.05. Fluo.: aggregates associated fluorescence. Individual points are experimental means, error bars show standard error of the mean

While most studies assessing axonal filtration in asymmetric microchannels have evaluated axonal outgrowth with only one compartment seeded, it has been shown that axons can serve as guides to neuronal processes growing in the opposite direction [33]. We thus chose to quantify axonal filtration in chimeric networks, where either the presynaptic or postsynaptic compartment was seeded with hippocampal primary neurons expressing the fluorescent protein tdTomato (Hip^mTmG+/–^) neurons while the other compartment was seeded with non-fluorescent (Hip^mTmG−/−^) neurons (Fig. 1c). This allowed us to selectively observe either forward or backward axonal outgrowth in reconstructed neural networks (Fig. 1d and e). Axon valves completely prevented backward axonal outgrowth while allowing forward axonal outgrowth and robust invasion of the postsyn compartment (Fig. 1f, Supp Fig. 2).

### Active anterograde *trans*-neuronal transfer of exogenous aSyn aggregates in reconstructed neural networks decreases with maturation

aSyn aggregates are taken up by neurons and transported anterogradely and retrogradely in their axons [18, 19, 30]. We used our novel experimental setup to quantitatively demonstrate neuron-to-neuron transfer of aggregated aSyn from donor to recipient naïve neurons and subsequent seeding in the latter. We locally exposed reconstructed neural networks to two distinct and well-characterized recombinant human aSyn aggregates, Oligomers (hOlig) and Fibrils (hFib). Those differ in size, structural and biochemical characteristics [15], and have been shown to disseminate with different efficiencies in vivo, hOlig inter-neuronal transfer being the most efficient [34]. The exogenous aggregates were covalently bound to the atto 647 fluorophore, which allowed us to follow their fate in the culture. In order to get a robust quantification of aggregates-associated fluorescence, we developed an image analysis method which automatically delineated neuronal somas from phase migrographs, permitting the quantification of somatic fluorescence in many individual neurons (up to several hundred) in each culture system.

We first evaluated if exogenous aggregates were transferred from presyn to postsyn neurons through axonal projections. We used two types of networks: either both compartments were seeded with Hip neurons (Hip>Hip), or only the postsyn compartment was seeded (Ø>Hip). We introduced medium containing 500 nM of hFib or hOlig (concentration of aSyn monomers) in the presyn compartment for 24 h starting at day in vitro 14 (DIV14) and monitored the presence of exogenous aggregates colocalized with postsyn neuronal somas 3 days later, at DIV17 (Fig. 2a). Autofluorescence was observed in control untreated networks in the form of somatic puncta and could not readily be visually distinguished from weak aggregate-associated fluorescence in postsyn neurons of treated networks (Fig. 2b). We thus quantified the presence of fluorescent aggregates in neuronal somas by measuring the mean fluorescent signal in somas and normalizing all results by measurements in somas of untreated networks. For both types of aggregates, we observed a ~ 10% increase in postsyn neurons fluorescence in treated Hip > Hip networks, while we observed no increase in postsyn fluorescence in treated Ø > Hip networks (Fig. 2c). Because the length of the microchannels only allows axons to grow through [35], this demonstrates that hFib and hOlig do not passively diffuse between the presyn to the postsyn compartments, but are instead actively and anterogradely transferred by axonal projections.

The propagation of aSyn aggregation in neural networks is often considered “trans-synaptic”, despite the absence of data conclusively demonstrating the role of synaptic structures [22, 23]. Thus, the role of synaptic structures in the transfer of aSyn aggregates remains uncertain. Indeed, aSyn assemblies can be secreted in the extracellular medium by axonal projections [18] and traffic between neurons in immature networks which are not synaptically connected [19]. We thus sought to address the impact of synaptic maturity on aSyn aggregates propagation. In our culture system, axonal projections from presyn neurons progressively invade the postsyn compartment, and trans-compartment synaptic connectivity increases between DIV14 and 21 in Hip>Hip networks (Supp Fig. 3), as we previously observed in cortico-striatal networks [36]. We hypothesized that if aSyn aggregates propagation is favored by the presence synaptic structures, their transfer rate would be higher in more mature and synaptically connected Hip > Hip networks.

**Fig. 3:**
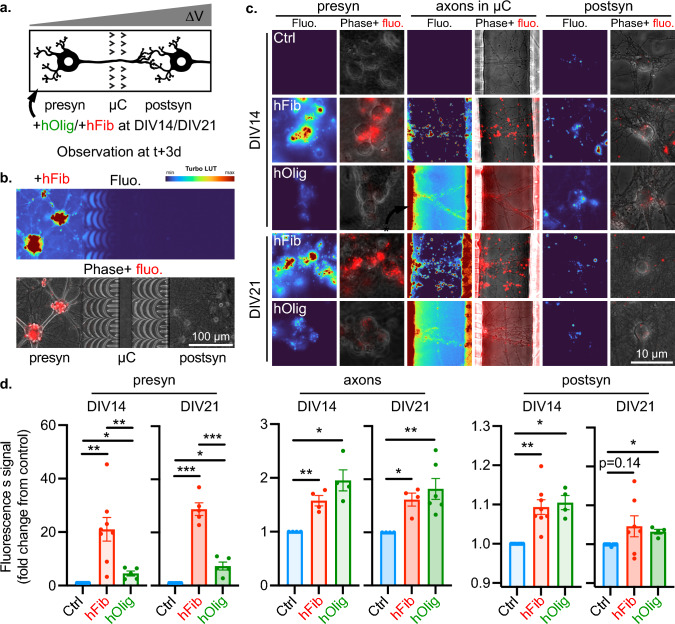
Exogenous human aSyn Fibrils and Oligomers are anterogradely transferred from neuron to neuron in immature and mature neural networks. **a** Schematic representation of the experimental design. The presynaptic compartment of Hip>Hip networks was spiked at DIV14 or DIV21 with control solution, 500 nM hFib or 500 nM hOlig, and aggregates associated fluorescence was monitored in neuronal somas in the postsyn and presyn compartments, and in axons in the central compartment 3 days later. **b** Low magnification epifluorescence microscopy field of a representative Hip > Hip network at DIV17, 3 days after presynaptic exposure to 500 nM hFib. Top, hFib and hOlig associated fluorescence is shown with the Turbo look-up table (LUT). Bottom, hFib and hOlig fluorescence is in red and Phase signal in gray. **c** Representative epifluorescence microscopy fields of presynaptic somas, axons in the central compartment and postsynaptic somas, 3 days after exposure of the presynaptic compartment to the indicated treatment. For each column, the left column highlights hFib associated fluorescence with the Turbo colormap, and the right column shows hFib fluorescence in red and Phase signal in gray. **d** Quantification of the aggregates associated fluorescence signal in neuronal somas in the presynaptic and postsynaptic compartments, and in axons in the central compartment. DIV14 presyn: *n* = 24–33, *N* = 5–8. DIV21 presyn: *n* = 22–26, *N* = 5–7. DIV14 axons: *n* = 15–19, *N* = 4. DIV21 axons: *n* = 14–19, *N* = 4–6. DIV14 postsyn: *n* = 16–54, *N* = 4–9. DIV21 postsyn: *n* = 22–42, *N* = 4–8. (*n* individual culture devices from *N* individual experiments). For comparing hFib and hOlig conditions to the control (normalized to 1 in each experiment), a one sample t-test against a hypothetical value of 1 was performed. For comparing hFib and hOlig conditions together, an unpaired t-test with Welch’s correction was performed. ***p < 0.001; **p < 0.01; *p < 0.05. Individual points are experimental means, error bars show standard error of the mean

To test this hypothesis, we exposed the presyn compartment of DIV14 or DIV21 Hip>Hip networks to 500 nM of hFib or hOlig for 24 h and monitored the distribution of aSyn aggregates 3 days after the initial introduction of aggregates in the system (Fig. 3a). The fluorescence of the media containing similar concentrations of hFib and hOlig differed by less than twofold (data not shown). While the spatial distribution of fluorescence remained strongly skewed toward the presyn compartment (Fig. 3b), we observed an increase in fluorescence in axons from presyn neurons and in the somas of postsyn neurons (Fig. 3c). We first quantified aggregate fluorescence in the somas of presyn neurons and observed that exposure to hFib resulted in a 21-fold increase in signal when spiking was performed at DIV14 and a 29-fold increase of fluorescence when it was performed at DIV21. hOlig uptake was significantly less efficient, with a 4.5-fold increase in fluorescence at DIV14 and a 7.3-fold increase at DIV21 (Fig. 3d). This is in good agreement with previous results from cultured neurons showing that hFib bind much better to neuronal membranes than hOlig [24]. Aggregates associated fluorescence was also observed in axons in the central compartment, with a 60% increase in signal in hFib treated networks at both DIV14 and DIV21, and a 95% and 80% increase in signal in hOlig treated network at DIV14 and DIV21, respectively (Fig. 3d). While hFib fluorescence was mostly contained within axonal projections in this compartment, a diffuse fluorescent signal could be observed in the axonal compartment of hOlig treated networks (Fig. 3c). This diffuse signal might be due to a small secretion of oligomers by axonal tracts [37–39] and passive diffusion to the central compartment.

We next quantified trans-neuronal transfer of exogenous aggregates. In DIV14 networks, both hFib and hOlig treatment resulted in a statistically significant 10% increase in fluorescence in postsyn neuronal somas. In DIV21 networks, despite higher aggregate fluorescence in presyn somas, the signal increased more modestly in postsyn somas, 4.6% and 3.2% upon hFib or hOlig treatment, respectively (Fig. 3d). To evaluate if the modest trans-neuronal transfer rate of aggregates was caused by an impact of microchannels geometry on neuronal physiology, we evaluated the health of axons grown through straight, 10 µm wide microchannels or through axon valves. We found that axonal fragmentation was similar in both conditions (Supp Fig. 4a and b). We also evaluated the impact of microchannels geometry on axonal aggregates transport and did not find that aggregates transport speed differed in the two microchannels geometries (Supp Fig. 4c and d). Thus, axon valves microchannels do not seem to affect axonal health when compared to standard straight microchannels. We then asked if the presyn and postsyn increase in global neuronal fluorescence could be mainly attributed to a sub-population of “super-uptakers”. We plotted the distribution of hFib and hOlig fluorescence in individual somas (Supp Fig. 5). The distribution of fluorescence appeared unimodal in presyn neurons and in postsyn neurons in treated networks. This suggests that neurons in hippocampal primary cultures bind aggregates with a relatively uniform efficiency, both when exposed to aggregates directly in the culture medium or through transfer by axonal projections.

**Fig. 4:**
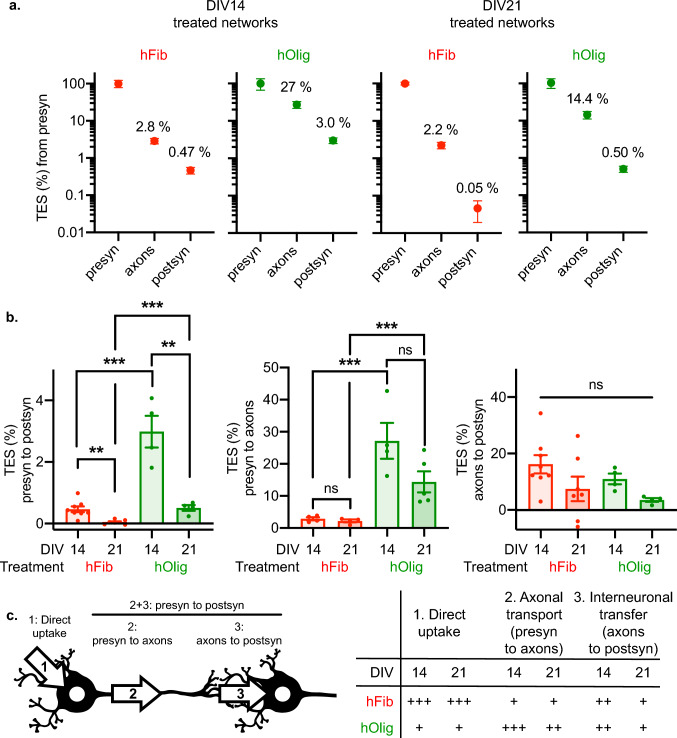
Oligomers are more efficiently transferred than Fibrils, and network maturity decreases the trans-neuronal transfer of aSyn aggregates. **a** Transmitted Excess Signal (TES) profiles generated from results reported in Fig. 4 (see “Methods” for calculation). Mean and standard error of the mean are shown in log scale. **b** TES in between each compartment. Two-way ANOVA followed by Tukey’s multiple comparisons test. ***p < 0.001; **p < 0.01; *p < 0.05. **c** Summary of observed transmission rates in between each compartment. Left, scheme of the different steps of an axon dependent trans-neuronal transfer of exogenous aggregates. Right, summary of the efficiency (from +/– : inefficient to +  +  + : efficient) of the different steps depending on the nature of the exogenous aggregate and the maturity of the network. Those ratings of relative efficiencies are derived from the TES quantifications in panel **b**. Step 1 refers to the two rightmost graphs in Fig. 4(**d**). Error bars show standard error of the mean

Because the quantity of signal associated with presyn neurons significantly differed between hFib and hOlig exposed networks, we sought to selectively quantify the efficiency of aggregates transfer from the somatodendritic compartment of presyn neurons to presyn axons and postsyn somas. We expressed our result as “Transmitted Excess Signal” (TES): a metric which expresses the fluorescent aggregates signal in the axonal or postsyn compartments as a percentage of the signal detected in the presyn or axonal compartment above background fluorescence (see “Methods” for details). We plotted TES for hFib and hOlig as a function of culture maturity (Fig. 4a). The TES from presyn to postsyn is higher for hOlig than for hFib, with respectively 3% and 0.47% of aggregates transferred in DIV14 networks, and 0.5% and 0.05% transferred in DIV21 networks (Fig. 4b). This suggests that hOlig are more efficiently transferred inter-neuronally than hFib. The presyn to postsyn TES of both hOlig and hFib were lower at DIV21 than DIV14, suggesting that increased network maturity decreased the inter-neuronal transfer of aSyn aggregates, despite increased synaptic connectivity (Supp Fig. 3).

In conclusion, these data support that following fixation to presynaptic somas, hOlig are more efficiently transported to postsynaptic neurons than hFib. This is to some extent expected and in good accordance with previous observations made in vivo [34]. In addition, contrary to our expectations, the transfer of both types of aggregates from presyn to postsyn neurons decreased with network maturation and increased synaptic connectivity (Fig. 4c).

### Anterograde*** trans-***neuronal propagation of aSyn seeding is inefficient and starts in the axons of postsynaptic neurons

Amplification of pathological aggregates through seeding of soluble aSyn is thought to drive the long term and long-distance propagation of aggregation in brain networks. However, neural network topology alone does not predict the path of aggregate propagation in brain networks, and the efficiency of retrograde versus anterograde aggregates spread remains elusive [28]. We thus sought to evaluate the efficiency of anterograde trans-neuronal propagation of aSyn aggregation.

**Fig. 5:**
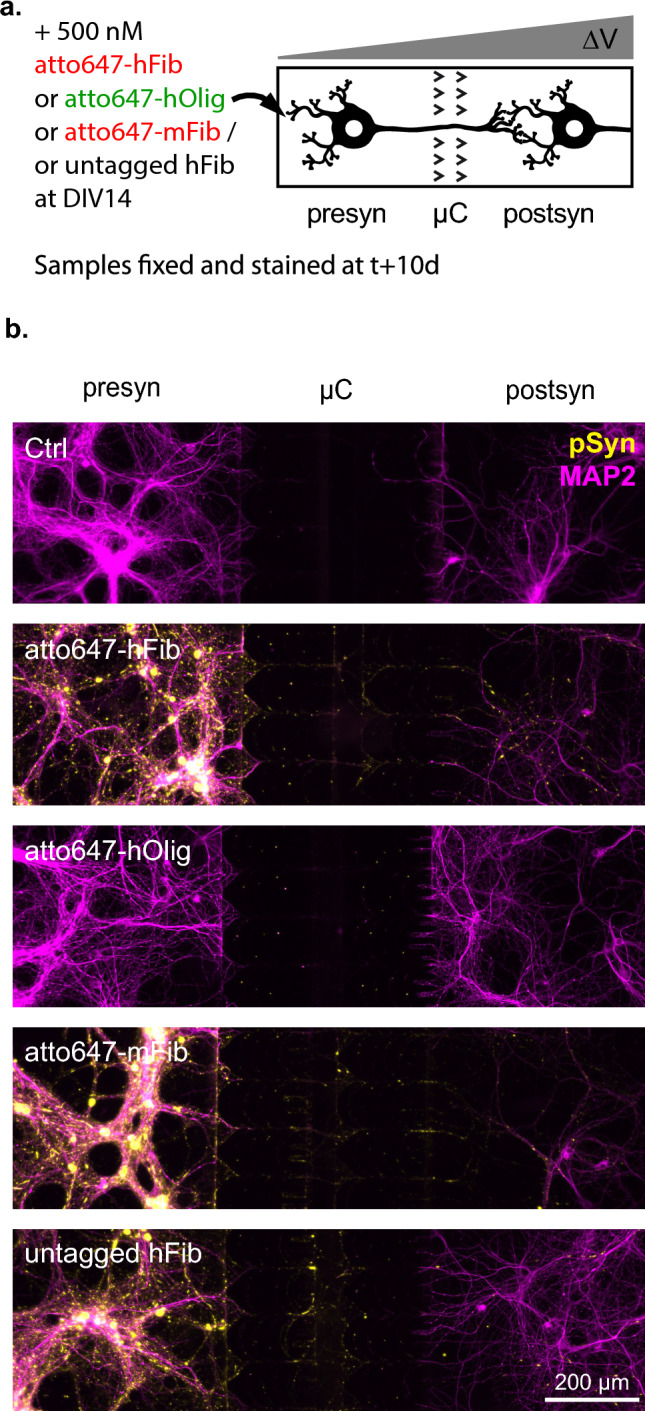
Endogenous aSyn aggregation is detected in the presynaptic and postsynaptic compartments but not in postsynaptic somas following presynaptic exposure of a Hip>Hip network to Fibrils. **a** Schematic representation of the experimental design. The presynaptic compartment of Hip>Hip networks were spiked at DIV14 with control solution, 500 nM atto647 tagged human aSyn Fibrils (atto647-hFib), 500 nM atto647 tagged human aSyn Oligomers (atto647-hOlig), 500 nM atto647 tagged murine aSyn Fibrils (atto647-mFib) or 500 nM untagged human aSyn Fibrils (untagged hFib). The cultures were fixed and stained 10 days later. **b** Representative epifluorescence fields of Hip>Hip networks after exposure to the indicated treatment. MAP2 staining is shown in magenta, pSyn in yellow

**Fig. 6:**
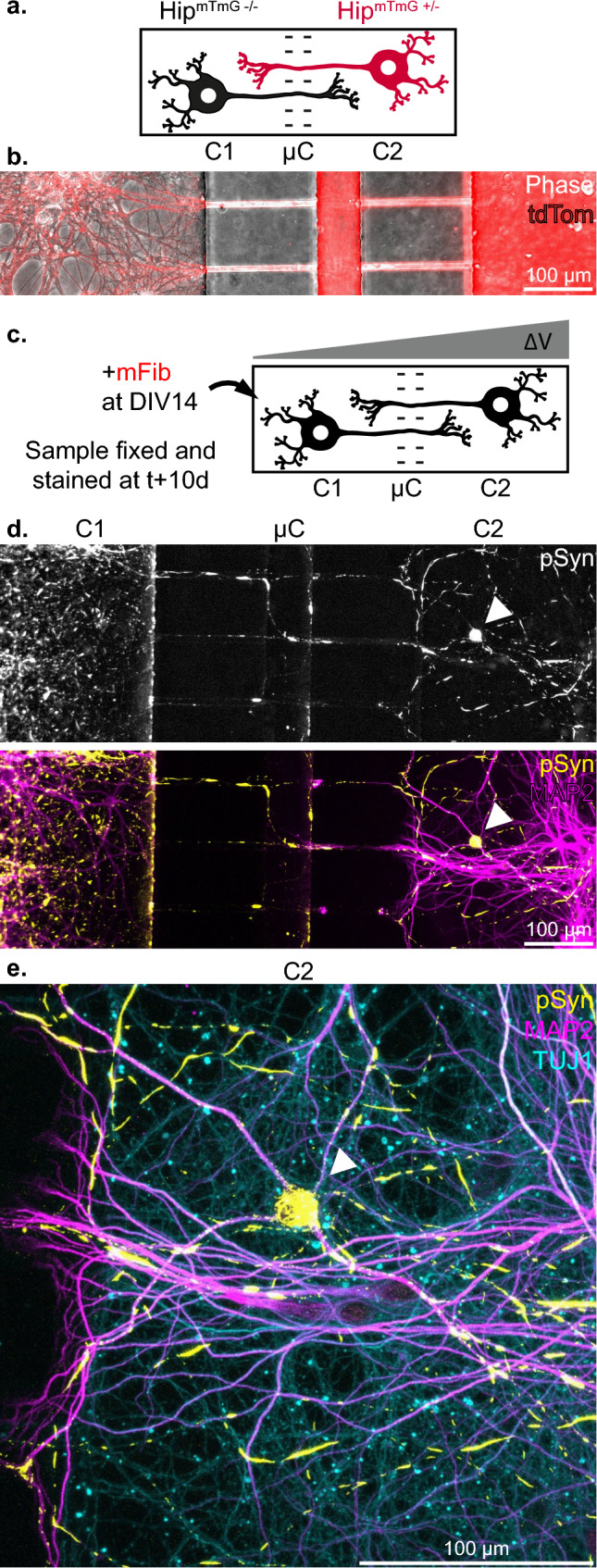
Endogenous aSyn aggregation propagates to the soma of secondary neurons in bidirectional Hip-Hip networks. **a** Schematic depiction of a Hip^mTmG−/−^–Hip^mTmG+/–^ bidirectional network. Straight 10 µm wide microchannels, in a device otherwise similar to the unidirectional network culture system in other aspects, allow axons to grow in both directions between culture compartments. **b** Representative image of axonal tracts connecting the two culture compartments in a Hip^mTmG−/−^–Hip^mTmG+/–^ bidirectional network. **c** Schematic representation the experimental design. The compartment 1 (C1) of a Hip-Hip network was spiked at DIV14 with 500 nM of mFib. A pressure gradient was maintained during the whole culture after spiking to prevent passive diffusion to the second compartment (C2). The culture was fixed 10 days later. **d** Representative image of a Hip-Hip network treated as explained in (**c**). The white arrow points to a C2 neuron containing numerous somatic pSyn inclusions. **e** Confocal imaging of the neuron highlighted in (**d**), highlighting the unambiguous somato-dendritic accumulation of pSyn inclusions

We previously showed selective phosphorylation of Ser 129 residue upon seeded aggregation of endogenous aSyn [40, 41]. To observe the seeding of endogenous aSyn by exogenous aSyn aggregates, the presyn compartment of DIV14 Hip>Hip networks was exposed to 500 nM of atto647-tagged hFib, hOlig, murine aSyn Fibrils (mFib) or untagged hFib for 24 h, fixed and stained by anti-Ser 129 phosphorylated aSyn (pSyn) antibody 10 days later (Fig. 5a). Exposure to all three types of Fibrils, but not hOlig led to the formation of numerous pSyn positive inclusions in the three compartments (Fig. 5b). When no neurons were present in the presyn compartment, no pSyn staining was observed in the postsyn compartment (Supp Fig. 6). This supports that seeding species do not diffuse passively between compartments in the 10 days following initial spiking, given that a pressure gradient is maintained in the microfluidic system. It has been reported by others that in a tri-compartmented microfluidic culture system with straight microchannels permitting bidirectional axonal growth, exposure of a peripheral compartment leads to the development of intracellular aSyn inclusions in neuronal somas two compartments away [42]. In our unidirectional networks, we observed no somatic inclusions in postsyn neurons in over 20 hFib treated Hip>Hip networks carefully examined by epifluorescence and confocal microscopy. In contrast, introduction of hFib on one side of bidirectional compartmentalized Hip-Hip networks led to the accumulation of somatic pSyn inclusions in 1–3 second-order neurons per network, in *n* = 10 networks from *N* = 2 separate experiments (Fig. 6). This suggests that previously reported trans-neuronal propagation of aggregation in triple compartmentalized networks might either result from a higher efficiency of retrograde trans-neuronal propagation, or as observed here, from the direct exposure of axonal projections from secondary neurons.

**Fig. 7:**
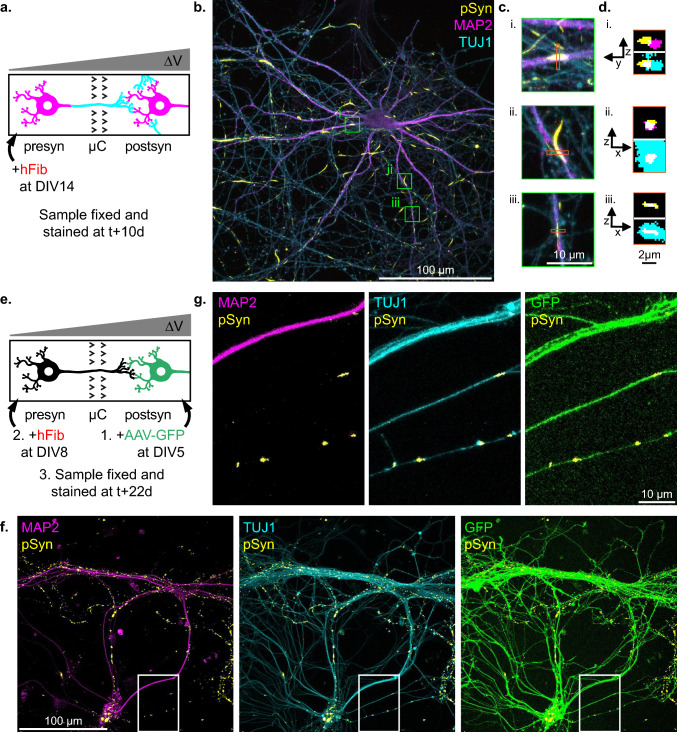
Exposure of the presynaptic compartment of reconstructed networks to aSyn Fibrils leads to modest and purely axonal aSyn aggregation in postsynaptic neurons. **a** Schematic representation of the first experimental design. The presynaptic compartment of Hip>Hip networks was spiked at DIV14 with 500 nM hFib, and cultures were fixed and stained 10 days later. **b** Representative confocal microscopy field of a postsynaptic neurons 10 days after hFib spiking of the presynaptic compartment. pSyn inclusions localization is ambiguous and appears mostly axonal. **c** Higher magnification view of the fields highlighted in (**b**), showing on pSyn inclusions that appear totally (i., iii.) or partially (ii.) colocalized with MAP2. **d** (*x*, *z*) or (*y*, *z*) view of the regions highlighted in (**c**). It appears that pSyn inclusions which do not appear to be contained in neuritic projections (ii.) colocalizes as much or more with the MAP2 signal than inclusions which appear to be contained in MAP2 from the (x,y) view. **e** Schematic representation of the second experimental design. To determine if some of the pSyn inclusions are located in the axons of postsynaptic neurons, the postsynaptic neurons of Hip>Hip networks were exposed to AAV-GFP at DIV3, days before axons reach from the presynaptic to the postsynaptic compartment. The networks were then processed as previously. **f** One of the rare confocal microscopy fields in which an unambiguous colocalization of GFP and pSyn signals was observed. **g** Magnification of the region from fields in (**f**) containing the unambiguous co-localization of GFP, pSyn and TUJ1 signals in isolated axonal projections

While we observed no somatic inclusions in postsyn neurons, we hypothesized that inclusions might first form in neuritic projections, as neuropathological observations suggest [43]. Indeed, the subcellular localization of pSyn rich inclusions in the postsyn compartment of Hip>Hip networks was ambiguous. Confocal imaging revealed that pSyn inclusions colocalized with axons. Colocalization of pSyn with MAP2 could not be attributed with certainty to seeding in the secondary neurons and might instead be due to the fasciculation of axonal and dendritic processes (Fig. 7a–d). In order to determine if some of the axonal inclusions that we detected in the postsyn compartment were located in axons from the postsyn neurons, we selectively infected the postsyn population with an adeno-associated viral vector encoding the enhanced green fluorescent protein (AAV-GFP) (Fig. 7e). While the interpretation of most GFP-pSyn colocalizations were made ambiguous by the fasciculation of numerous axonal projections from both presyn and postsyn axons, colocalization of pSyn inclusions with isolated GFP positive axons was unambiguous. However, we could detect this unambiguous colocalization in only ~ 25% of individual reconstructed networks 10 days following exposure of the presyn neurons to hFib (Fig. 7f and g). These data support that anterograde transfer of aSyn seeds from presynaptic neurons leads is slow, of the order of a few pM per day per axon (calculation in the Discussion section).

### Presynaptic neuronal subtypes do not affect exogenous aggregates transfer

**Fig. 8:**
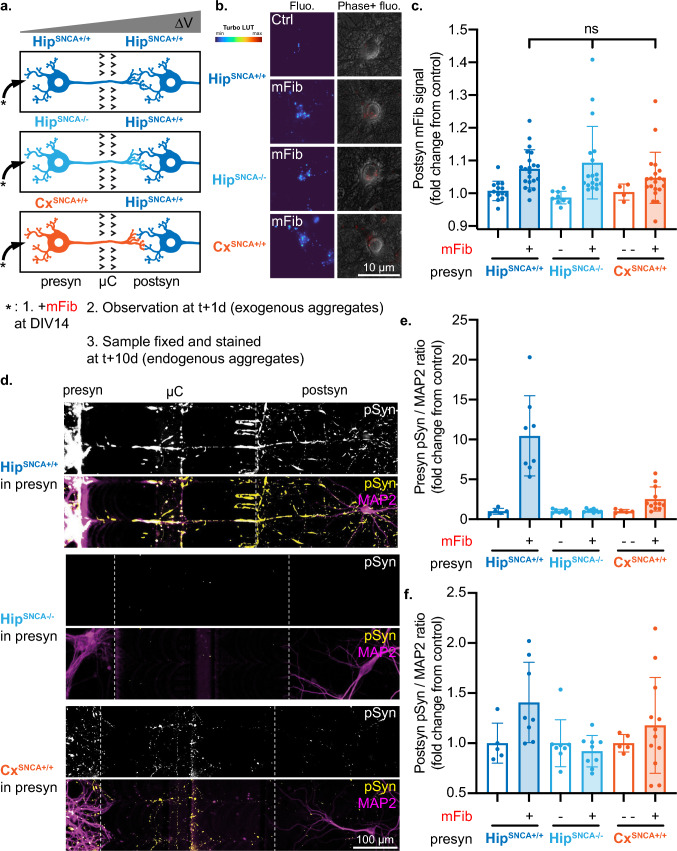
Presynaptic identity does not impact fast aggregates transfer but does impact endogenous aSyn aggregates seeding. **a** Schematic representation of the experimental design. The presynaptic compartment of Hip^SNCA+/+^>Hip^SNCA+/+^, Hip^SNCA−/−^>Hip^SNCA+/+^ and Cx^SNCA+/+^>Hip^SNCA+/+^ networks was spiked at DIV14 with 500 nM mFib, aggregates associated fluorescence was monitored in neuronal somas in the postsyn compartment 3 days after treatment, and networks were fixed and stained 10 days after treatment. **b** Representative epifluorescence microscopy fields of postsynaptic neurons 1 day after presynaptic exposure to mFib. Left, mFib associated fluorescence is shown with the Turbo colormap. Right, mFib fluorescence is in red and Phase signal in gray. **c** Quantification of mFib associated fluorescence in postsynaptic neurons. Results from individual culture devices are shown as individual data points. Kruskall-Wallis test followed by Dunn’s multiple comparisons test was performed selectively on data from mFib treated networks. *n* = 18–21 devices from *N* = 3 individual experiments in mFib treated networks, and *n* = 4 to 14 devices from *N* = 3 individual experiments in ctrl networks. **d** Representative epifluorescence microscopy fields of stained networks, 10 days after presynaptic exposure to mFib. **e**, **f** Quantification of the pSyn/MAP2 signals ratio in **e** presynaptic and **f** postsynaptic compartments. *n* = 5–12 culture devices from *N* = 1–2 individual experiments. Error bars show standard deviation

aSyn aggregation propagates within the brain of patients in distinct neural networks, and some have suggested that the characteristics of neuronal populations making up the networks impact the efficiency of rate-limiting steps in this propagation, independently of the synaptic strength between those populations [26–28]. Supporting this notion, we previously established a relationship between the seeding propensity of exogenous murine aSyn Fibrils (mFib) and endogenous aSyn expression level using distinct neuronal cultures (hippocampal, cortical and striatal primary mouse neurons, wild-type or aSyn knockout) [40]. To determine whether the nature of the neuronal population also impacts exogenous aSyn aggregates propagation, we reconstructed networks where we changed the identity of the presynaptic populations: hippocampal (Hip^SNCA+/+^), cortical (Cx^SNCA+/+^) or aSyn knockout hippocampal (Hip^SNCA−/−^) cultures, resulting in the following chimeric networks: Hip^SNCA+/+^>Hip^SNCA+/+^, Hip^SNCA−/−^>Hip^SNCA+/+^ and Cx^SNCA+/+^ > Hip^SNCA+/+^ (Fig. 8a). We treated the presyn compartment of these networks at DIV14 with 500 nM mFib and followed aggregates transfer to postsyn neurons 1 day after treatment. In this set of experiments, we decided to use mFib instead of hFib, as we had previously characterized their seeding efficiency in the neuronal populations of interest, and wanted to assess the relationship between seeding and transfer efficiency [40]. We observed an increase in mFib associated fluorescence in postsyn neurons in the three types of networks considered (Fig. 8b). The increase in fluorescence from background signal was of 8%, 7% and 4% in Hip^SNCA+/+^>Hip^SNCA+/+^, Hip^SNCA−/−^>Hip^SNCA+/+^ and Cx^SNCA+/+^>Hip^SNCA+/+^, respectively. Transfer efficiency did not differ significantly between treated networks, even when mean fluorescence measurements in individual culture devices were used for statistical analysis instead of experimental means (Fig. 8c). We next assessed pSyn accumulation 10 days after treatment. In the presyn compartment, as previously published [40], we found that pSyn signal was absent in Hip^SNCA−/−^, low in Cx^SNCA+/+^, and high in Hip^SNCA+/+^ neurons. The quantity of pSyn in the postsyn compartment reflected the quantity of pSyn detected in presyn (Fig. 8d, e, f). However, this signal appeared to be mostly contained in axonal projections from presyn neurons, and we did not observe any somatic pSyn inclusions in postsyn neurons in any of the 29 treated networks. Careful examination of the 9 Hip^SNCA−/−^>Hip^SNCA+/+^ networks treated with mFib did not reveal any trace of pSyn in postsyn axons and dendrites.

These results suggest that Hip^SNCA+/+^, Hip^SNCA−/−^ and Cx^SNCA+/+^neurons transfer exogenous aSyn fibrils to postsynaptic neurons at comparable rates. They also support that in this experimental timeframe, the vulnerability of presyn neurons to aSyn seeding does not radically impact the propagation of aggregation to postsyn neurons. Those data contrast with previous reports showing faster exogenous aSyn aggregate propagation in aSyn knockout as compared to WT mice [44].

## Discussion

Pathological aSyn spreading is widely considered “trans-synaptic” given that regions that sequentially develop LB are most often synaptically connected and that neuroanatomical connectivity supports the spread of LB better than a nearest neighbor scheme [45, 46]. aSyn assemblies have been shown to traffic between neuronal cells after release in the culture medium naked or within exosomes [47], through tunneling nanotubes and gap junctions [17, 48, 49], and possibly immature synapses [19]. Thus, while the cellular and molecular mechanisms underlying inter-neuronal transfer remains unclear, quantifying the efficiency of seeds propagation between synaptically connected neurons is important for both accurately modeling the spread of aSyn aggregates and pinpointing biomolecular cues involved in that transfer. Here, using a newly developed microfluidic device allowing the reconstruction of fully oriented binary neuronal networks with virtually no retrograde connections, we demonstrate that the prion-like dissemination of aSyn aggregates between two synaptically interconnected neurons is a slow process, and that different types of aSyn aggregates are transferred with distinct efficiencies. This slow anterograde transfer leads to the exposure of postsyn neurons to low doses of seeding aggregates, resulting in a modest seeding of endogenous aSyn aggregation which starts in axonal projections. This is indeed compatible with the progression of Parkinson’s disease over years or decades.

Our results provide a first quantitative estimate of exogenous aSyn aggregates anterograde transfer between interconnected neurons. While aSyn aggregates are efficiently taken up by neurons, most exogenous aggregates remain within the somato-dendritic compartment, with only ~ 2% of the Fibrils found within axonal shafts of presynaptic neurons. Fibrils and Oligomers were not transferred with the same efficiency to postsynaptic neurons. 0.5% of presyn hFib were detected in postsynaptic neurons 3 days after presynaptic inoculation in DIV14 networks, while this percentage was higher for Oligomers, at around 3%. This is in agreement with previous observations made in vivo showing that aSyn Fibrils spread to second order neurons to a much lesser extent than monomeric or aSyn Oligomers after delivery within the olfactory bulb [34, 50].

We sought to roughly estimate the rate at which exogenous fluorescent aggregates were transported from neuron to neuron in our system. Liberally assuming that there is a linear correlation between fluorescence increase and aggregate exposure in the medium, we calculated the approximate concentration of aggregates which should be directly introduced in the postsyn compartment to lead to a similar fluorescence increase. For calculations simplicity, we also assumed that aggregates-associated fluorescence was not affected by axonal transport, transfer to secondary neurons, or bleaching, though we can not rule out those phenomena.

Three days after presynaptic exposure of Hip>Hip networks to 500 nM of hFib, the fluorescence increase in the postsynaptic compartment corresponded to a direct exposure to approximatively 1–10 nM and 100–1000 pM of hFib in DIV14 and DIV21 networks, respectively. In good agreement with our previous observation that direct exposure of Hip cultures for 14 days results in a predominantly axonal distribution of pSyn staining [40], we observed the same staining distribution in postsyn neurons 10 days after exposure of presyn neurons to 500 nM of hFib (Fig. 7). With an average of ~ 100 axons innervating the postsyn compartment, and assuming a constant aggregate transfer rate, in this setup a single presyn neuron would anterogradely transfer enough hFib to expose postsyn neurons to around 5–50 pM per day. This rate falls to ~ 0.5–5 pM of hFib per day in DIV21 networks. In the same experimental setup, the rate of anterograde hOlig transfer per axon is of ~ 10–100 pM/day in DIV14 networks and ~ 5–50 pM/day in DIV21 networks. Those calculations do not indicate the exact amount of aggregates which are transferred by axon: this value could be obtained, for example, by using aggregates made from individually labelled aSyn proteins. We instead provide a rough and indirect order of magnitude for the rate of anterograde inter-neuronal transfer of aSyn aggregates, which might help guide the parametrization of computational models.

Surprisingly, the transfer of both hFib and hOlig was lower in DIV21 networks compared to DIV14 networks even though synaptic connectivity between the two populations increased with time of culture. This suggests that in our experimental conditions and in the considered timeframe, maturation of synaptic structures does not facilitate the inter-neuronal transfer of aSyn aggregates. This is in good agreement with previous observations that synaptic connectivity alone does not predict the propagation of aSyn aggregation in the human brain [28].

Moreover, in our experimental paradigm neither the synapse identity (cortex vs hippocampal) nor the genetic status (WT vs aSyn knockout) of presynaptic structures significantly modified trans-neuronal spreading efficiency.

Previous works aiming at investigating aSyn aggregates prion-like propagation within in vitro reconstructed neural networks suffered from several limitations, such as an absence of synaptic connectivity [19], or uncertainty as to the direction of axonal growth in the first generation of microfluidic devices [42, 51]. The results we report here, using a novel microfluidic culture system to generate synaptically mature and fully unidirectional neural networks in which the trans-neuronal transfer of aSyn aggregates can unequivocally be attributed to active anterograde axonal transport, are in sharp contrast with reports of efficient trans-neuronal spreading in networks reconstructed with conventional non-filtering microfluidic devices [42, 51]. Some of those previous results have since then been contradicted [52]. The difference in propagation efficiency we observed in our system compared to systems of the previous generation might arise from direct exposure of axons originating from neurons in the distal chamber of the microfluidic device that invade the proximal chamber, where aSyn aggregates are added. This is strongly suggested by our results showing significant endogenous aSyn seeding in secondary neurons grown in microfluidic devices with straight micro-channels. While this calls for attention upon use of microfluidic systems, this also evidences direct uptake of aSyn by axonal termini and very efficient retrograde transfer of aSyn seeds towards neuronal cell bodies. A partial correlation between synaptic connectivity and the pattern of propagation of aSyn aggregation was recently evoked [26–28]. Our results suggest that direct axonal capture of injected aggregates may partly or fully account for these observations.

While our results show that increased network maturity accompanied by increased synaptic connectivity does not increase interneuronal aggregates transfer, many aspects of cultured neurons physiology other than synaptic connectivity evolve in the weeks following plating and might modify transfer efficiency:

First, changes in the neuronal transcriptome and neuronal physiology (longer and more arborized neurites) might affect binding, uptake, seeding and clearance of exogenous aSyn aggregates.

Second, immature neurons exhibit higher exocytic and endocytic activities, which might result in heightened uptake and secretion of exogenous aggregates, and could explain why transfer is more efficient in young networks [53]. To note, while our data show a trend of higher uptake of hFib and hOlig in mature cultures compared to immature ones (Fig. 3), this difference was not statistically significative (as assessed by an unpaired t-test with Welch's correction).

Third, astrocytes proliferate over time in neuronal cultures and they have been shown to take up and degrade exogenous aSyn assemblies [54], thus diverting aSyn assemblies transfer from neuron-neuron to neuron-astrocytes in older cultures. However, we observed that aggregates direct uptake was not diminished by culture maturation, arguing against a higher rate of aggregates capture by astrocytes with culture maturation (Fig. 3).

Fourth, we did not characterize how synaptic morphology (postsynaptic dendritic spine morphology and molecular identity) differs between DIV14 and 21 cultures. Some synaptic structures might be more permissive to interneuronal aggregates transport.

Fifth and last, neuronal activity might affect potential aggregates trafficking at synaptic junctions as well as other process. While neuronal activity is known to affect the rate of axonal transport of vesicles, as well as the rate of vesicles exocytosis and the association of aSyn to synaptic vesicles [55], a recent study suggested that increased network maturity leads to a progressive slowdown of axonal transport, thus possibly impacting aSyn aggregates transport to the postsynaptic compartment [56]. Hip>Hip networks reconstructed in our microfluidic devices exhibit spontaneous, correlated neuronal activity evidencing functional connections between the culture chambers (calcium imaging data not shown). This suggests that the trans-neuronal transfer occurred in synaptically active networks. Acute modulation of synaptic activity with biccuculine or tetrodotoxin at the presynaptic level did not affect the amount of *trans*-neuronal transfer (data not shown). Yet, in our view, this does not preclude a role of synaptic activity in aSyn aggregates spreading. However, we believe that this question is beyond the scope of this paper. Indeed, chronic modification of neuronal activity leads to profound synaptic homeostatic changes which in turn lead to reversal of enhanced activity trough presynaptic and postsynaptic homeostatic plasticity processes [57, 58]. Therefore, while of great interest, studying the consequences of chronic modification of synaptic activity would require the validation of a protocol for chronically modifying neuronal activity over several days in vitro (e.g. through the use of stimulating microelectrode array with patterned currents), which is not a trivial matter.

aSyn prion-like dissemination in brain networks theoretically encompasses two sequential steps involving (1) trans-neuronal spread of aSyn seeds and (2) amplification of seeds through a templated nucleation process. In previous studies we demonstrated a seeding efficiency dependence on seeds concentration and structural characteristics, and on endogenous aSyn expression in recipient cells [40, 59]. A recent analysis of *in vivo* datasets has shown that tau seeds propagation in the brain is best described by a replication-limited model, in which local replication of the seeds, while slow, dictates the pattern of propagation [60]. Consistent with those results, we found that while we could detect the trans-neuronal spreading of aSyn seeds in a matter of days, the multiplication of those seeds in secondary neurons, indirectly evidenced by pSyn staining, was much slower. Moreover, while we previously found that aSyn expression strongly affected the seeding of endogenous aSyn aggregation in different neuronal populations [40], the rate of trans-neuronal seeds transfer from those populations did not statistically differ. This emphasizes that there is some decorrelation between the dissemination of seeding aggregates in neural networks and the buildup of inclusions made of endogenously produced aSyn. Those data thus support that the stereotypical patterns of aSyn aggregation in brain networks could be mostly driven by difference in local aSyn seeds replication rate, rather than difference in seeds spreading efficiency between neuronal populations.

Direct exposure of neurons to hFib or mFib led, after 10 days, to an important accumulation of pSyn aggregates both at the somato-dendritic level and in the distal axonal shafts. Detecting pSyn aggregates in postsynaptic neurons turned out to be difficult as most of pSyn structures in post synaptic chambers belonged to the presynaptic axons. Nevertheless, using viral vectors to trigger GFP expression in postsynaptic neurons, we were able to detect a small amount of endogenous aSyn inclusions in postsynaptic neurons. Strikingly, all inclusions we detected were located in the distal axons of post synaptic neurons with no sign of pSyn accumulation in their somato-dendritic compartments. These results are in line with previous data showing that, in vivo, LB accumulation starts in branched axons and follows a “centripetal” pattern towards the nucleus in neurons [23, 27, 61, 62]. These observations have been consistently reproduced in primary neuronal cultures [40, 63]. The limited amounts of pSyn in postsynaptic neurons axons might be the consequence of the amount of hFib transferred to the second order neurons. In Hip>Hip DIV14 networks, 3 days after exposure of the presyn compartment to 500 nM of hFib, 0.5% of the signal in presyn neurons could be detected in postsyn neurons. Assuming a linear relationship between the concentration of hFib in culture medium and the fluorescence associated with neuronal somas, this suggests that postsyn neurons bound roughly as many aggregates as if they were directly exposed to at a dose of 2.5 nM of hFib in the medium.

aSyn expression level has been demonstrated to predict the vulnerability of neurons to seeding following direct exposure to exogenous aggregates [26, 40]. However, it remains unclear how neuronal identity affects the transfer of exogenous aggregates to secondary neurons. By exposing the presyn compartment of three types of chimeric networks to aSyn Fibrils, we found that while aSyn expression level did predict the endogenous aSyn seeding in presyn neurons, neuronal identity did not significantly impact transfer of Fibrils to postsyn neurons.

While we were unable to formally compare the rate of accumulation of pSyn in postsynaptic neurones between Hip^SNCA−/−^>Hip^SNCA+/+^ and Hip^SNCA+/+^>Hip^SNCA+/+^ networks due to previously stated technical limitations, the absence of pSyn signal in the postsyn compartment of Hip^SNCA−/−^>Hip^SNCA+/+^ networks indirectly suggests that amplification of aSyn aggregates in the presynaptic neurons might increase the likelihood of seeding in postsynaptic neurons. We believe that this specific point should be addressed by a specific set of experiments, maybe relying on a sensitive reporter of aggregation in postsyn neurons, such as aSyn fused to the moieties of a split fluorescent protein.

The vulnerability to seeding of a given neuron in a brain network depends on the membrane proteins it expresses that bind seeds, endogenous aSyn expression level, the rate of seeds uptake and clearance by the recipient neuron [40, 64] but also, and most important, on the release of seeds from affected neighbor neurites and somas. Overall, our results suggest that aSyn aggregates trans-neuronal spread depends on two distinct rate limiting steps encompassing (1) a size-dependent trans-neuronal traffic of aSyn aggregates and (2) a dependence on endogenous aSyn concentration leading to axon-first aSyn aggregation in second-order neurons. These aspects of aSyn aggregation might reveal important for interpreting LB distribution in vivo and the apparent discrepancy between LB accumulation and the connectome. Indeed, if one was to count all (dendritic, somatic and axonal) inclusions in a given brain region to determine its vulnerability to PD, the presence of axonal afferents from affected neurons which somas are located in another region might lead to an overestimation of the vulnerability of the neurons in this region.

## Conclusion

In this work, we exposed the design of a new compartmentalized neuronal culture system. This new system is the first that allows to reconstruct in vitro neural networks which are simultaneously unidirectional and synaptically connected, thanks to the use of a new generation of asymmetric microchannels we termed “axon valves”. It is thus the first to enable the quantitative study of the pure anterograde transfer of seeding and non-seeding aggregates in neural networks.

Thanks to the properties of our experimental system, we were able to address original questions regarding the biology of aSyn aggregation propagation. (1) We obtained a quantitative estimate of the rate of trans-neuronal transfer of aggregates per axon, which lies between 0.5 and 100 pM/day in our experimental setting. (2) By taking advantage of the inherent progressive synaptic maturation of reconstructed networks, we found that network maturity and increased synaptic connectivity did not facilitate the inter-neuronal transfer of aggregates. (3) We obtained experimental evidence that anterograde trans-neuronal transfer of seeding aggregates leads to a slow, axon-first seeding of endogenous aSyn in postsynaptic neurons. (4) We were able to decouple the impact of neuronal identity on fast exogenous aSyn aggregates inter-neuronal transfer from seeding of endogenous aSyn aggregation.

While the present study focused on aSyn aggregates, we believe the technology we describe here can be used in a versatile manner to study the anterograde trans-neuronal propagation of other neuronal pathogens and prion-like proteins.

## Methods

### Microfluidic culture chips

The structured part of the system, made of polydimethylsiloxane (PDMS), is cured on a mold fabricated in house by two-steps micro photolithography. It is then covalently bound to a glass coverslip. Three compartments, 50 µm in height, are sequentially linked by two arrays of 3 µm height, 175 µm length axon-permissive microchannels (Fig. 1a, Supp Fig. 1). While the two peripheral compartments, 1 mm wide, are dedicated to neuronal cultures, the central 50 µm wide compartment serves to observe axonal outgrowth between the two microchannels arrays and to improve fluidic isolation between the two culture compartments. It has indeed previously been demonstrated that when a proper pressure gradient is maintained in microchannels of such dimensions, small molecules do not diffuse from the lower pressure compartment to the other [35].

A detailed version of the protocol used for the fabrication of SU-8 master mold, resin mold and culture chips can be found in [29]. Briefly, microchannels and culture compartments were drawn using QCAD. The precise dimensions of the chips with axon valves microchannels are indicated in Supp Fig. 1. In experiments involving chips with straight microchannels, all dimensions were identical, except for the 10 axon valves microchannels that were replaced by 10 straight 10 µm wide microchannels. Photomasks engraved with the designs were ordered from Selba. Photosensitive negative SU-8 resin (MicroChem) was spin-coated to the desired height on a silicon wafer (Prolog Semicor) and exposed to UV light through a photomask encoding the culture chip outlines using an MJB4 mask aligner (Suss). Unexposed SU-8 was washed away. These steps were repeated twice, once with a low viscosity resin for 3 µm high microchannels, and once with a higher viscosity resin for 50 µm high culture compartments. The height of the mold was then checked with a stylus profiler (Dektak), and microstructures integrity was verified with an optical microscope (Leica). The resulting mold was used to cast polydimethylsiloxane (PDMS Sylgard, Ellsworth Adhesives) mixed at a 10:1 w:w ratio of base to curing agent. PDMS was cured 3 h minimum at 70 °C. For high throughput chip production, a sturdier epoxy resin mold was used. The resulting chips were unmolded, and inlets were punched with a surgical biopsy punch (4 mm diameter) at both extremities of each compartment. The PDMS blocks were bonded to 130–160 µm thick glass coverslips (Fisher Scientific 11,767,065) after plasma surface treatment with an Atto plasma cleaner (Diener). Culture compartments were then immediately filled with deionized water. Devices were placed in individual Petri dishes for easier handling, and each of the resulting culture system was sterilized by exposure to a UV lamp for 30 min. The day before cells seeding, culture devices were coated with Phosphate Buffered Saline (PBS, Thermo Fisher 14,190,169) containing 10 µg/mL poly-d-lysine (Sigma P7280), introduced in one well per culture compartment. Devices were then incubated overnight in a 37 °C humidified atmosphere. 4–6 h before cells seeding, wells were emptied, then filled in the same fashion with PBS containing 2.5–5 µg/mL laminin (Sigma L2020).

### Primary neuronal cultures

Animal care was conducted in accordance with standard ethical guidelines (U.S. National Institutes of Health publication no. 85–24, revised 1985, and European Committee Guidelines on the Care and Use of Laboratory Animals) and the local, IBPS and UPMC, ethics committee approved the experiments (in agreement with the standard ethical guidelines of the CNRS “Formation à l′Expérimentation Animale” and were approved by the “C2EA -05 Comité d’éthique en experimentation animale Charles Darwin”).

Hippocampal (Hip) and cortical (Cx) regions were micro-dissected from embryos from pregnant mice at embryonic day 16 (E16). Wild type (WT, Hip^SNCA+/+^) cultures were prepared from inbred Swiss mice (Janvier). Hip cultures knockout for aSyn (Hip^SNCA−/−^) were prepared from inbred C57bl/6Jolahsd mice (Envigo). Hip cultures expressing membrane associated tandem Tomato (tdTomato, Hip^mTmG+/–^) or not (Hip^mTmG−/−^) were obtained by cross-breeding C57bl/6 J *mTmG* +/– (adapted from [65]) males with C57bl/6 J *mTmG –/–* females. A fluorescent protein flashlight (Nightsea DFP-1) was used to distinguish *mTmG* +/– from *mTmG –/–* embryos.

Cx and Hip microstructures were washed in Gey’s Balanced Saline Solution (Sigma G9779) and chemically digested with papain (Sigma 76,220) diluted (7.5 units/mL for Cx, 15 units/mL for Hip) in Dulbecco's Modified Eagle Medium (DMEM, Thermofisher 31,966 021) at 37 °C. After digestion was interrupted by adding 10% Fetal Bovine Serum (FBS, GE Healthcare), supernatant was replaced with DMEM containing 40 units/mL of DNase type IV (Sigma D5025). Physical cell dissociation was performed by gently flowing microstructures 10 times through a 1 mL micropipette tip. Cell suspension was centrifuged (80 g, 8 min) and resuspended with DMEM. Cells concentration was assessed in a Malassez counting device. Cells were centrifuged again (80 g, 8 min) and suspended at the desired concentrations in culture medium (Neurobasal medium (Thermofisher 21,103,049) supplemented with 1% GlutaMAX (Thermofisher 35,050), 1% B27 Supplement (Thermofisher 17,504–044)) supplemented with 10% FBS. The central compartment of microfluidic devices was filled with culture medium before cells seeding, to prevent cells from flowing through microchannels during cells seeding. 1.5 µL of cells suspension at 40 and 10 million cells/ml were introduced in the presynaptic and postsynaptic compartments, respectively, and were left to adhere for 15 min. Culture wells were filled with culture medium supplemented with 10% FBS. Water containing 0.5% ethylenediaminetetraacetic acid (EDTA, Sigma EDS) was added in the Petri dish, around the chip, to limit evaporation. Culture devices were then placed in a humidified atmosphere at 37 °C and 5% carbon dioxide. Culture medium supplemented with 10% FBS was completely replaced 24 h after cell seeding by FBS-free culture medium. Half of the medium was replaced every week with fresh FBS-free culture medium.

### Immunostaining

For all steps, one inlet by compartment was filled to ensure solution flow though the culture compartment. Cultures were fixed at room temperature for 10 min in PBS containing 4% paraformaldehyde (Electron Microscopy Science 15714S) and 4% sucrose (Sigma S9378). Membrane permeabilization and saturation were performed simultaneously at room temperature for 30 min in PBS containing 0.2% Triton X-100 (Sigma X100) and 1% bovine serum albumin (BSA, Thermofisher 15,561,020). Primary and secondary antibodies were diluted in PBS 1% BSA and were respectively incubated 16 h at 4 °C and 1 h at room temperature. A list of antibodies used in this study can be found in Supplementary Table 1.

### Image acquisition

Epifluorescence imaging was performed using a DMi8 microscope (Leica) fitted with a pE-4000 LED light source (CoolLED) and a sCMOS Flash 4.0 camera (Hamamatsu). A 40X NA 0.8 objective was used for high magnification imaging, and a 10X NA 0.4 objective for low magnification imaging.

Confocal imaging was performed with a SP5 set-up (Leica) fitted with a 63X NA 1.4 objective.

### aSyn aggregates generation

Mouse (m) or human (h) aSyn proteins were produces as described in (Ghee et al. 2005 FEBS J). Human aSyn Oligomers (hOlig) were obtained as described in [16]. Mouse and human fibrils (mFib and hFib, respectively) were assembled as described in [15]. The nature of aSyn assemblies was assessed by Transmission Electron Microscopy (TEM) after adsorption of the aggregates onto carbon-coated 200 mesh grids and negative staining with 1% uranyl acetate using a Jeol 1400 transmission electron microscope. The images were recorded with a Gatan Orius CCD camera (Gatan, Pleasanton, CA, USA). aSyn oligomers and fibrils were fluorescently labeled in PBS buffer with ATTO 647 sulfo-NHS dye at a dye-protein ratio of 1:1 following manufacturer recommendation (ATTO-Tech, Gmbh) and as previously described [24]. Fibrillar aSyn was fragmented by sonication for 20 min in 2-ml Eppendorf tubes in a Vial Tweeter powered by an ultrasonic processor UIS250v (250 W, 2.4 kHz; Hielscher Ultrasonic, Teltow, Germany) to generate fibrillar particles with an average size 42–52 nm as assessed by TEM analysis. Aliquots (5 µl at 350 µM, concentration of monomers) of aSyn aggregates were flash frozen in liquid nitrogen and stored at − 80 °C. Samples were thawed in a 37 °C water bath on the day of culture treatment.

### Spiking of neuronal cultures with aSyn aggregates

Cultures in microfluidic devices were first selected based on cellular integrity in both compartments, and robust axonal outgrowth from presynaptic to postsynaptic compartment. Treatment of the presynaptic compartment with hFib, hOlig of mFib was performed at DIV13-14 or 20–21. Culture wells were completely emptied, and inlets of the postsynaptic, central and presynaptic compartments were filled sequentially, respectively with 45, 35 and 10 µL of medium. Postsynaptic and central inlets were filled with fresh culture medium, and presynaptic inlets were filled with culture medium containing or not 500 nM of freshly thawed aggregates, thoroughly mixed with culture medium by pipetting up and down 30 times. To ensure that presynaptic neurons were homogeneously exposed to aggregates, 2.5 µL of the bottom inlet were transferred to the top inlet. 24 h after treatment, the inlets of the presynaptic compartment were emptied, and neurons were rinsed one time by filling one well with 15 µL fresh medium, waiting 1 min and then emptying both inlets. Both presynaptic inlets were then filled with 15 µL of fresh medium.

### Image analysis and quality control before analysis

Image analysis was performed using custom image analysis pipelines for Fiji version 2.0.0 [66]. The main steps of each analysis are explained below.

A first time before treatment, and a second time before analysis, cultures were selected on the basis of cell survival and robust axonal outgrowth to the second compartment. These parameters were determined from the Phase channel. In our experimental timeframe, we did not observe a lower survival in cultures treated with 500 nM hOlig, hFib or mFib in comparison to control ones.

Aggregates associated fluorescence was shown in all figures using the look-up table (LUT) Turbo, obtained from https://github.com/cleterrier/ChrisLUTs.

Specific neuronal somas selected for illustrating figures were selected on the basis of the measurement of their fluorescence falling very close to the average somatic fluorescence for the condition they illustrate.

### Analysis of axonal filtration

Crossing tdTomato positive axons in Hip^mTmG+/–^ > Hip^mTmG−/−^ and Hip^mTmG−/−^ > Hip^mTmG+/–^ networks were manually counted at the exit of microchannels in presynaptic or postsynaptic compartments from epifluorescence microscopy 10X fields.

### Analysis of synaptic density

Cultures were stained with anti-Bassoon, MAP2 and beta-III-tubulin antibody. aSyn staining was additionally performed in Hip^SNCA+/+^ > Hip^SNCA−/−^ networks. Confocal Z-stacks were acquired with a 0.42 µm z step and 0.24 × 0.24 µm pixels. Z-stacks were then submitted to maximum intensity projection. The Bassoon channel was pretreated with a 5-pixel radius Subtract Background, while the aSyn channel was pretreated with a 20-pixel radius Subtract Background. Fields to be analyzed were selected based on low dendrites density and high axonal density. Isolated dendrites traces were drawn from the MAP2 channel using the Simple Neurite Tracer plugin [67] on Fiji. Traces were used to determine neurite length and were dilated to 3 µm width regions of interest (ROIs). ROIs were then used to segment the sections of the Bassoon channel to be analyzed. The Trainable WEKA Segmentation plugin [68] on Fiji was trained to recognize Bassoon foci, and applied to all Bassoon channels. Probability maps were binarized. Particles larger than 1 pixel were numbered with Analyze Particles to generate individual Bassoon ROIs. For determining the density of aSyn + synapses, the mean aSyn signal was measured in each Bassoon ROI. Synapses were then classified as aSyn + or aSyn –  by an arbitrary threshold on the aSyn mean intensity. A minimum of 650 µm of dendrites from at least four separate fields were considered in each individual culture compartment.

### Analysis of aSyn aggregates axonal transport

Neuronal cultures in compartmentalized microfluidic culture systems separated by axon valves or straight, 10 µm wide microchannels (same length, height and distance as axon valves) were exposed to 500 nM of atto647 tagged hFib at DIV8 and rinsed at DIV9. Time lapse imaging was then performed at DIV13. Micrographs were acquired every 5 s for 5 to 10 min with a 10X objectives, the field of view contained from 6 to 7 microchannels. Kymographs were then generated from ROIs traced in individual microchannels between the central and postsyn compartments. Particle paths were manually counted to quantify the percentage of moving particles. To quantify particles speed, paths were manually traced following straight tracks in the kymographs, ignoring particle pausing. Those tracks were then used as individual data points for quantifying the mean particle speed in technical replicates, and for plotting the distribution of particle speed in those individual paths.

### Analysis of aSyn aggregates fluorescence in somas

For analyses involving exogenous fluorescent aggregates, five frames were taken in Z with a step of 2 µm, and analysis was performed on the maximum intensity Z-project of these frames. Cultures had to be imaged live, as aggregates associated fluorescence decreased drastically after fixation. Thus, neuronal somas had to be detected from the Phase channel. ROIs circling neuronal somas were automatically detected from the Phase channel using a modified version of the region-convoluted neural network (R-CNN) described in [69], available at the following https://github.com/matterport/Mask_RCNN. The algorithm was trained over 150 iterations using 4000 ROIs of neuronal somas manually traced in 350 fields in both presynaptic and postsynaptic compartments. Parameters were adjusted to the size of the desired ROIs. Automatically generated ROIs were then manually validated and corrected before segmentation of fluorescence channels. The mean aggregates associated fluorescence was measured in each ROI. Results were normalized by the mean fluorescence in untreated cultures imaged during the same microscopy session. A minimum of 40 somas from at least 4 separate fields were considered in each individual culture compartment.

### Analysis of aSyn aggregates fluorescence in axons

Individual axon tracts in the central channel of culture devices were manually traced using the Phase channel. Analysis of the fluorescence channel was then performed in a 1 µm wide ROI surrounding manually traced axonal tracts. Results were normalized by the mean fluorescence in untreated cultures imaged during the same microscopy session. A minimum of 10 axons were considered in each individual culture compartment.

### Calculation of transmitted excess signal

Transmitted Excess Signal (TES) in individual experiments was computed using the following equations:

$${\mathrm{TES}}_{\mathrm{presyn}-\mathrm{postsyn}}=\frac{\left({S}_{\mathrm{postsyn}}-{S}_{\mathrm{background}}\right)}{\frac{1}{n} {\sum }_{1}^{n}\left({S}_{\mathrm{presyn} n}-{S}_{\mathrm{background}}\right)},$$

$${\mathrm{TES}}_{\mathrm{presyn}-\mathrm{axons}}=\frac{\left({S}_{\mathrm{axons}}-{S}_{\mathrm{background}}\right)}{\frac{1}{n} {\sum }_{1}^{n}\left({S}_{\mathrm{presyn} n}-{S}_{\mathrm{background}}\right)},$$

$${\mathrm{TES}}_{\mathrm{axons}-\mathrm{postsyn}}=\frac{\left({S}_{\mathrm{postsyn}}-{S}_{\mathrm{background}}\right)}{\frac{1}{n} {\sum }_{1}^{n}\left({S}_{\mathrm{axons} n}-{S}_{\mathrm{background}}\right)}.$$

With *S*_*i*_ as the average of normalized signals in compartment *i* in an individual experiment, *S*_background_ as 1 (because ctrl values cluster around 1 after normalization), and *n* the total number of experiments for which signal in the presyn compartment was obtained.

### Distribution of aSyn aggregates associated fluorescence in individual somas

Distributions of signal in 500 randomly picked individual somas per condition were obtained by plotting data with the distplot() function of the Seaborn package on Python 3 with 100 bins.

### Analysis of endogenous aSyn aggregates

S129 phosphorylated aSyn (pSyn) and MAP2 were stained. Integrated signal densities were measured in the whole presynaptic and postsynaptic compartments, imaged by epifluorescence microscopy with a 10X objective. pSyn signal was normalized by MAP2 signal. Results were normalized by the pSyn/MAP2 ratio in untreated cultures imaged during the same microscopy session.

### Infection with adeno-associated viral vectors

Primary neuronal cortical-hippocampal networks were cultured as previously described. At DIV5, hippocampal neurons of the postsyn chamber were transduced with AAV10 vectors (pssAAV-CBA-GFP-WPRE) with the dose of 10^8^ viral genomes/8.000 cells. At DIV8, the cell culture medium was replaced with fresh medium in order to eliminate the residual viral particles. Selective treatment of the postsyn compartment with viral vectors was performed in the same fashion than the spiking of the presyn compartment with aggregates described earlier. At DIV8, the presyn compartment was spiked with 500 nM mFib, as previously described. Cultures were then maintained for 22 days (17 days after AAV transfection at 14 days after aSyn exposure) at 37 °C in 5% CO_2_. They were then fixed and stained before imaging by confocal microscopy.

### Statistical analysis

Data analysis was performed with the Pandas package in Python 3. Statistical analysis and graphs generation were performed with GraphPad Prism version 8.4.2.

If not indicated otherwise, individual data points on graphs represent experimental means. Experimental means from at least two individual culture devices were used as individual data points for the purpose of statistical analysis.

First, the deviation from the normal (gaussian) distribution of each replicate in a condition was assessed with a Shapiro–Wilk’s test. Then, two-columns comparisons were performed with a t-test with Welch’s correction if both columns followed a normal distribution and with a Mann–Whitney test otherwise. Comparisons of more than two columns with one parameter varying were performed with a Brown-Forsythe ANOVA test followed by Dunnett’s T3 multiple comparisons test if all columns followed a normal distribution, and with Kruskal–Wallis test followed by Dunn’s multiple comparisons test otherwise. Comparisons of more than two columns with two parameters varying were performed with two-way ANOVA with the Geisser-Greenhouse correction followed by Tukey’s multiple comparisons test. In the case of Fig. 3d, where results in each experiments were normalized so that the average of the control was equal to 1, a one sample *t*-test was performed against a hypothetical value of 1 for comparing hFib and hOlig conditions to the control (all samples passed a normality test).

### Supplementary Information

Below is the link to the electronic supplementary material.

Supplementary Figure 1: Dimensions of axonal filtration microchannels. **a** Design of a single device for oriented network reconstruction. In grey: 50 µm high compartments, in white: 3 µm high microchannels. **b** Zoom on the microchannels. **c** Zoom on a single microchannel motif. Critical dimensions are highlighted. (PDF 1082 KB)

Supplementary Figure 2: Robust axonal invasion from the presyn to the postsyn compartment. **a** Epifluorescence microscopy field of a representative Hip^mTmG+/–^>Hip^mTmG–/–^ network at DIV19. Presynaptic neurons densely innervate the region in front of microchannels in the postsynaptic compartment. **b** Epifluorescence microscopy field of a representative Hip>Hip network at DIV24. (PDF 9516 KB)

Supplementary Figure 3: Synaptic connectivity between the presynaptic and postsynaptic populations increases with culture time. **a** Schematic representation of the experimental design. Hip^SNCA+/+^>Hip^SNCA–/–^ networks permitted the estimation of overall synaptic density by staining for synaptic proteins, and of inter-compartment synaptic structures by staining for the presynaptic aSyn protein. **b** Representative confocal microscopy field of neurons in the postsynaptic chamber of a 21 days old culture. White arrows highlight synaptic foci stained with both aSyn and VGLUT1. **c**–**e** Evolution of synaptic connectivity over culture time. **c** Representative confocal microscopy field of dendrites from the postsynaptic compartment of Ø>Hip^SNCA–/–^ and Hip^SNCA+/+^>Hip^SNCA–/–^ networks. **d** Quantification of the number of Bassoon foci per µm of dendrites in the postsynaptic compartment. Individual data points represent individual culture devices. **e** Quantification of the number of Bassoon foci also positive aSyn per µm of dendrites in the postsynaptic compartment. Individual data points represent individual culture devices. *n *= 3–5 individual culture devices from *N *= 1 individual experiment. Error bars show standard deviation. (PDF 3396 KB)

Supplementary Figure 4: Axonal health and hFib transport are not affected by axonal growth through axon valves. **a** Schematic representation of the experimental design for evaluating axonal fragmentation. Hip neurons were seeded in the presyn compartment of microfluidic culture systems separated by axon valves microchannels (µC) (“>” signs) or by straight, 10 µm microchannels of otherwise similar dimensions and spatial distribution as axon valves (“–” signs). Cultures were fixed at DIV13 and stained with TUJ1. **b** Representative micrographs of TUJ1 staining at the exit of microchannels with the fluorescent signal in inverted black and white. **c** Schematic representation of the experimental design for quantifying hFib axonal transport. Neuronal seeding was performed as in (**a**). Neuronal somas were then exposed to 500 nM atto647 tagged hFig at DIV8, rinsed at DIV9. Timelapse imaging was performed on the distal microchannels. **d** Data analysis was performed on kymographs generated from ROIs traced on the distal segment of microchannels. *n *= 6–7 culture devices from *N *= 1 individual experiment. Error bars show standard deviation. **e** Distribution of the probability density of absolute particle speeds on single straight tracks obtained from kymograph data. (PDF 3763 KB)

Supplementary Figure 5: Distribution of hFib and hOlig associated fluorescence in presynaptic and postsynaptic neurons. Histograms (100 bins) of the distribution of aggregates associated fluorescence in 500 somas randomly picked from *n *= 8–21 individual culture devices from *N *= 2–5 individual experiments. Somas located in the (**a**, **b**) presynaptic or (**c**, **d**) postsynaptic compartment of Hip>Hip networks treated at (**a**, **c**) DIV14 or (**b**, **d**) DIV21 with control solution (blue), 500 nM of hFib (orange) or 500 nM hOlig (green). (PDF 521 KB)

Supplementary Figure 6: Seeding aggregates do not passively diffuse between the culture compartments. **a** Schematic representation of the experimental design. The presynaptic compartment of Ø>Hip networks was spiked at DIV14 with 500 nM of mFib, and endogenous aSyn aggregation was monitored 10 days later. **b** Representative epifluorescence field of a Ø>Hip network. **c** Representative confocal microscopy field of the postsynaptic compartment of a Ø>Hip network. (PDF 2310 KB)

Supplementary File 7: Antibodies used in this study. (XLSX 9 KB)

## Data Availability

The datasets generated during the current study are available from the corresponding author on reasonable request.

## Abbreviations

AAV: Adeno Associated Vector
aSyn: Alpha-Synuclein
BSA: Bovine Serum Albumin
Cx: Cortical Primary Neurons
Cx > Hip: Cortical to Hippocampal Oriented Neural Network
DIV: Days In Vitro
DMEM: Dulbecco Modified Earl’s Medium
EDTA: Ethylenediaminetetraacetic Acid
FBS: Fetal Bovine Serum
GFP: Green Fluorescent Protein
hFib: Human-derived aSyn Fibrils
Hip^SNCA^^+/+^: Wild-Type Hippocampal Primary Neurons
Hip^SNCA^^−/−^: aSyn Knockout Hippocampal Primary Neurons
Cx^SNCA^^+/+^: Wild-Type Cortical Primary Neurons
Hip-Hip: Hippocampal to Hippocampal Bidirectional Neural Network
Hip: Hippocampal Primary Neurons
Hip>Hip: Hippocampal to Hippocampal Oriented Neural Network
Hip^mTmG +/−^: Hippocampal Primary Neurons Expressing tdTomato
Hi^mTmG +/−^: Hippocampal Primary Neurons not Expressing tdTomato
hOlig: Human-derived aSyn Oligomers
LB: Lewy Bodies
LUT: Look-Up Table
MAP2: Microtubule Associated Protein 2
mFib: Mouse-derived aSyn Fibrils
*N*: Number of experimental replicates
*n*: Number of technical replicates
NA: Numerical Aperture
ND: Neurodegenerative Diseases
nM: Nanomolar
Ø>Hip: Hippocampal Neurons Seeded Only in Receiving chamber
PBS: Phosphate Buffered Saline
PD: Parkinson’s Disease
PDMS: Polydimethylsiloxane
pM: Picomolar
Postsyn: Postsynaptic
Presyn: Presynaptic
pSyn: Ser129 Phosphorylated aSyn
ROI: Region Of Interest
tdTomato: Membrane-targeted Tandem Dimer Tomato
TES: Transmitted Excess Signal
t + Xd: X days post treatment
µC: Microchannels
µL: Microliter
µm: Micrometer
µM: Micromolar
