# Supplementary figures and images for: Synapses do not facilitate prion-like transfer of alpha-synuclein: a quantitative study in reconstructed unidirectional neural networks
Source: Cell Mol Life Sci. 2023 Sep 9;80(10):284. doi: 10.1007/s00018-023-04915-4 (PMC10492778; doi:10.1007/s00018-023-04915-4)

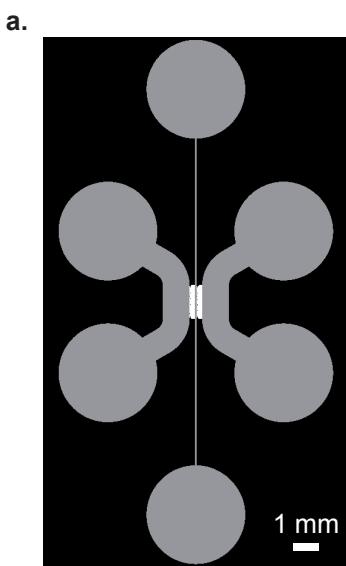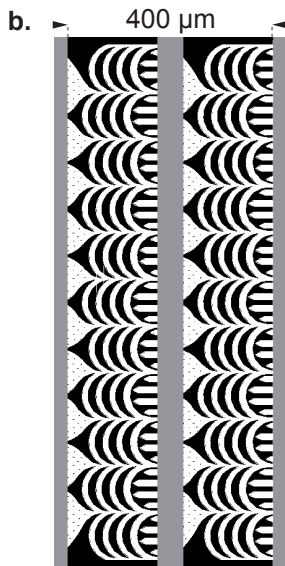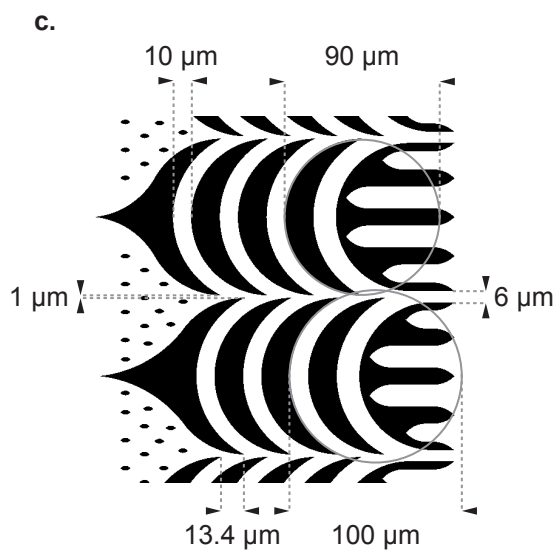

Supplement: Supplementary file 1 — Supplementary Figure 1: Dimensions of axonal filtration microchannels. a Design of a single device for oriented network reconstruction. In grey: 50 µm high compartments, in white: 3 µm high microchannels. b Zoom on the microchannels. c Zoom on a single microchannel motif. Critical dimensions are highlighted. (PDF 1082 KB) [file 18_2023_4915_MOESM1_ESM.pdf]

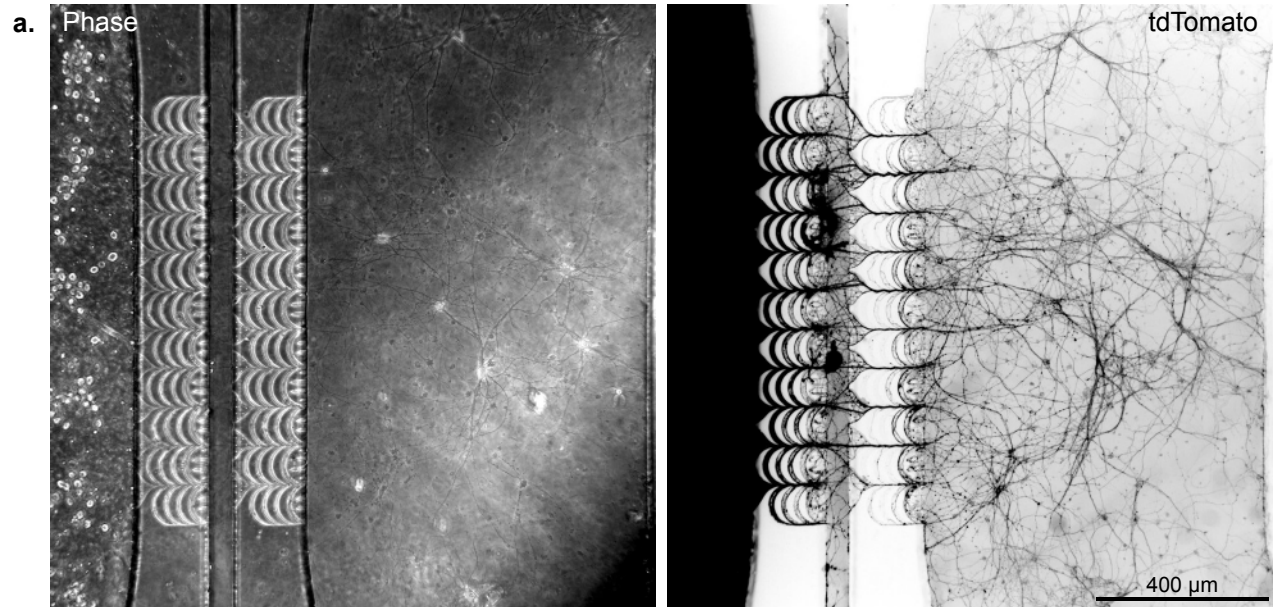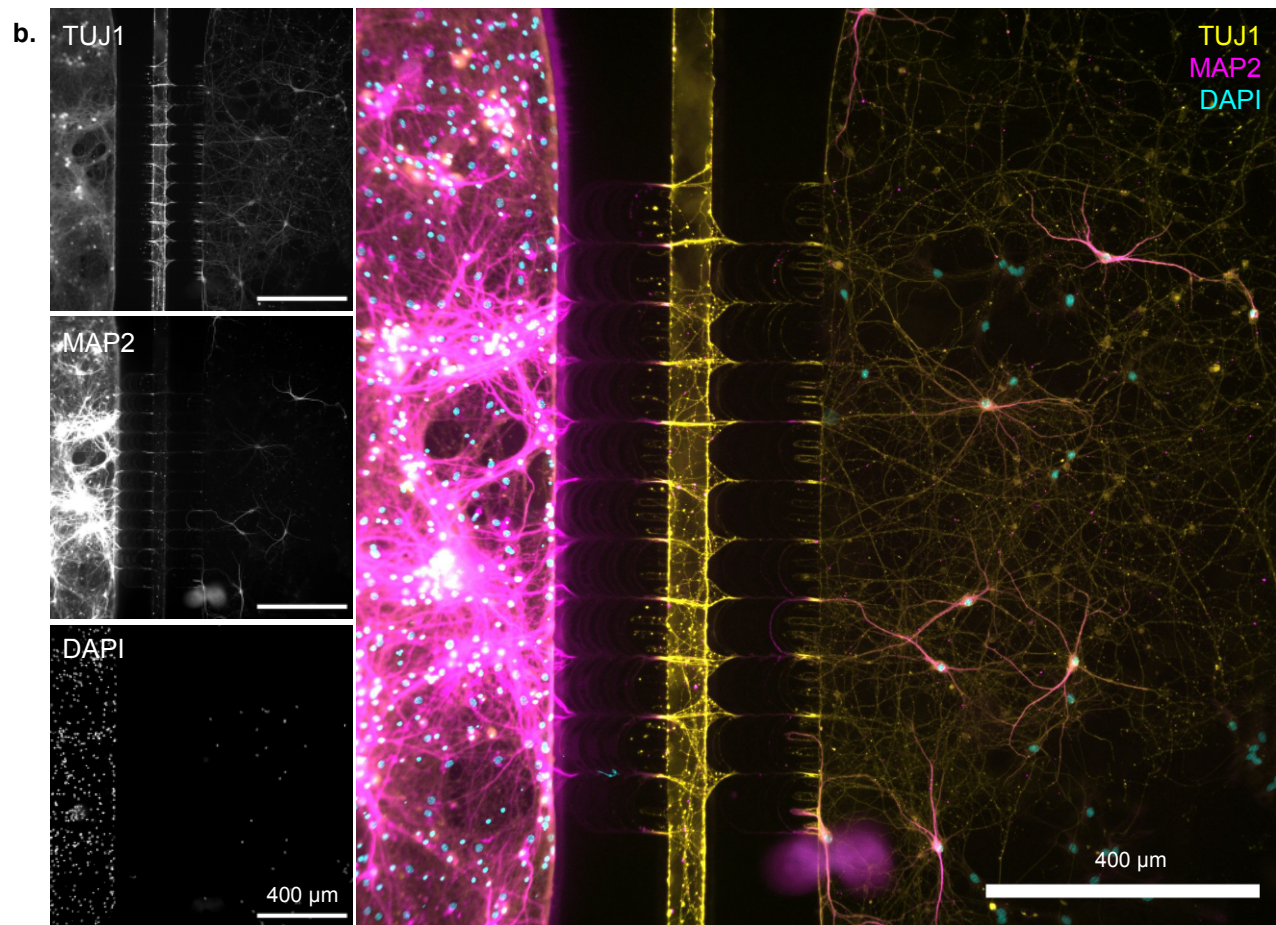

Supplement: Supplementary file 2 — Supplementary Figure 2: Robust axonal invasion from the presyn to the postsyn compartment. a Epifluorescence microscopy field of a representative HipmTmG+/–>HipmTmG–/– network at DIV19. Presynaptic neurons densely innervate the region in front of microchannels in the postsynaptic compartment. b Epifluorescence microscopy field of a representative Hip>Hip network at DIV24. (PDF 9516 KB) [file 18_2023_4915_MOESM2_ESM.pdf]

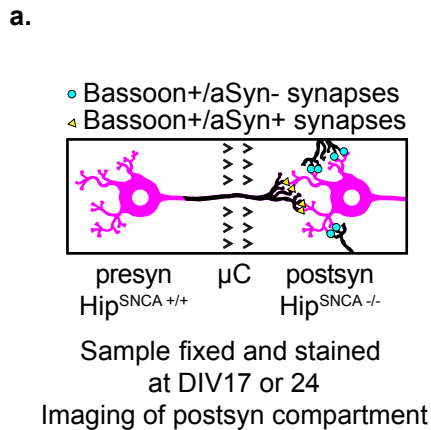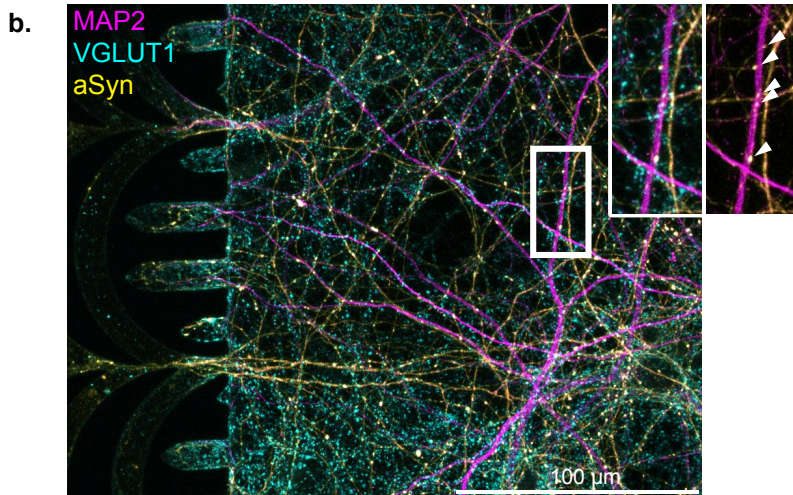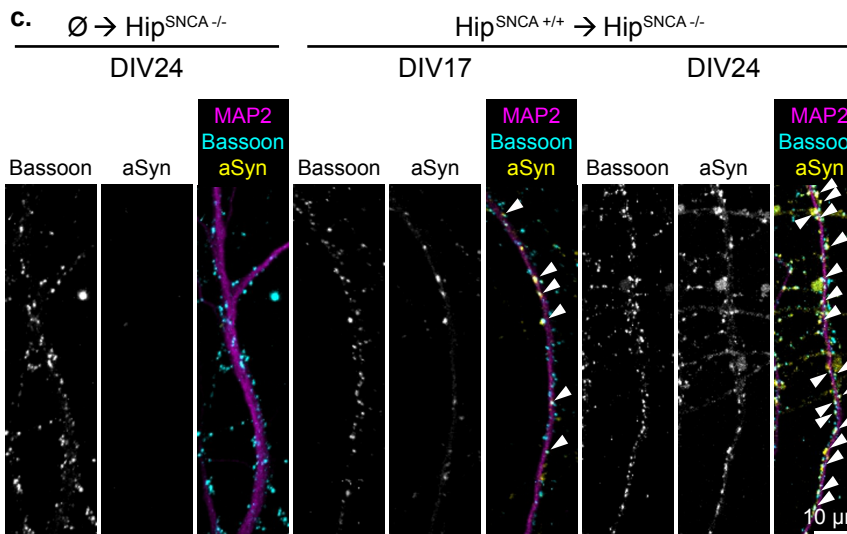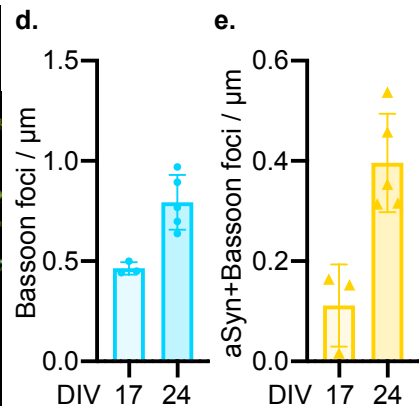

Supplement: Supplementary file 3 — Supplementary Figure 3: Synaptic connectivity between the presynaptic and postsynaptic populations increases with culture time. a Schematic representation of the experimental design. HipSNCA+/+>HipSNCA–/– networks permitted the estimation of overall synaptic density by staining for synaptic proteins, and of inter-compartment synaptic structures by staining for the presynaptic aSyn protein. b Representative confocal microscopy field of neurons in the postsynaptic chamber of a 21 days old culture. White arrows highlight synaptic foci stained with both aSyn and VGLUT1. c–e Evolution of synaptic connectivity over culture time. c Representative confocal microscopy field of dendrites from the postsynaptic compartment of Ø>HipSNCA–/– and HipSNCA+/+>HipSNCA–/– networks. d Quantification of the number of Bassoon foci per µm of dendrites in the postsynaptic compartment. Individual data points represent individual culture devices. e Quantification of the number of Bassoon foci also positive aSyn per µm of dendrites in the postsynaptic compartment. Individual data points represent individual culture devices. n = 3–5 individual culture devices from N = 1 individual experiment. Error bars show standard deviation. (PDF 3396 KB) [file 18_2023_4915_MOESM3_ESM.pdf]

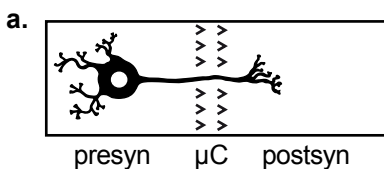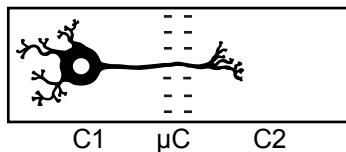

Fixed + stained at DIV13  
postsyn / C2 imaged

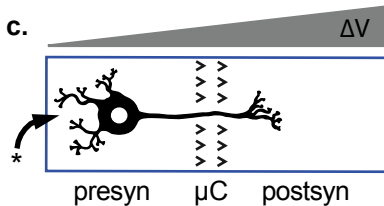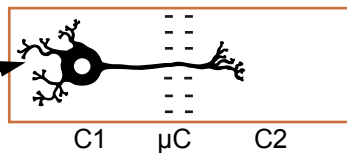

\* : +hFib at DIV8  
 $\mu$ C imaged at DIV13

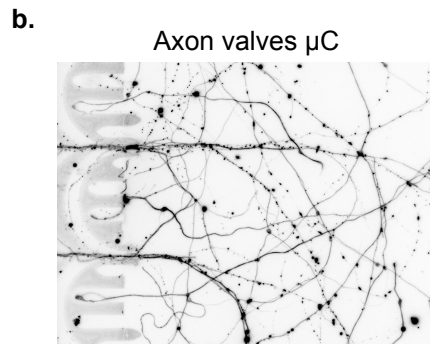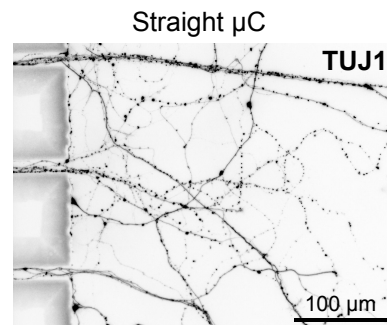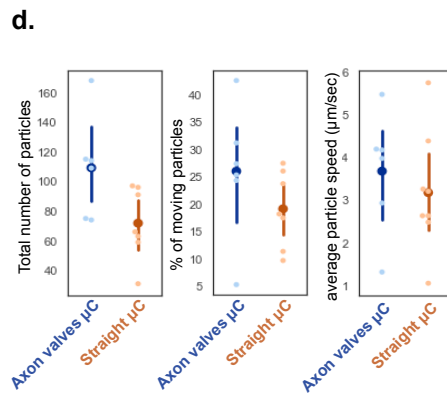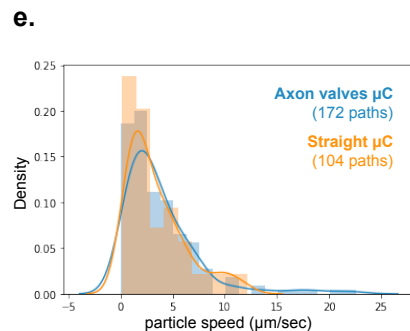

Supplement: Supplementary file 4 — Supplementary Figure 4: Axonal health and hFib transport are not affected by axonal growth through axon valves. a Schematic representation of the experimental design for evaluating axonal fragmentation. Hip neurons were seeded in the presyn compartment of microfluidic culture systems separated by axon valves microchannels (µC) (“>” signs) or by straight, 10 µm microchannels of otherwise similar dimensions and spatial distribution as axon valves (“–” signs). Cultures were fixed at DIV13 and stained with TUJ1. b Representative micrographs of TUJ1 staining at the exit of microchannels with the fluorescent signal in inverted black and white. c Schematic representation of the experimental design for quantifying hFib axonal transport. Neuronal seeding was performed as in (a). Neuronal somas were then exposed to 500 nM atto647 tagged hFig at DIV8, rinsed at DIV9. Timelapse imaging was performed on the distal microchannels. d Data analysis was performed on kymographs generated from ROIs traced on the distal segment of microchannels. n = 6–7 culture devices from N = 1 individual experiment. Error bars show standard deviation. e Distribution of the probability density of absolute particle speeds on single straight tracks obtained from kymograph data. (PDF 3763 KB) [file 18_2023_4915_MOESM4_ESM.pdf]

**a.**

DIV14

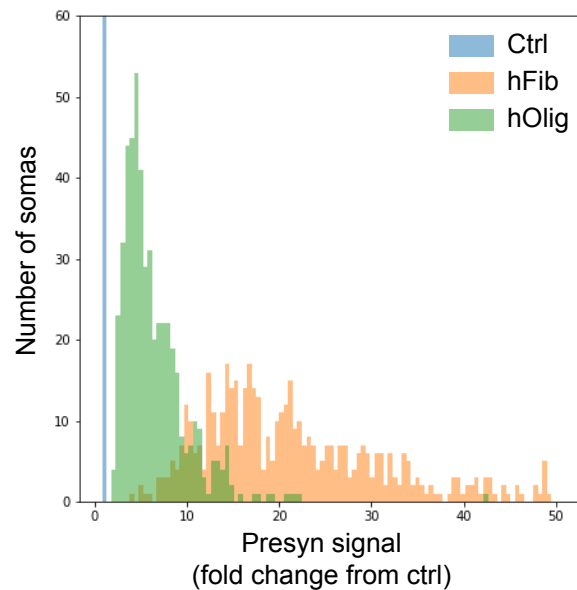**b.**

DIV21

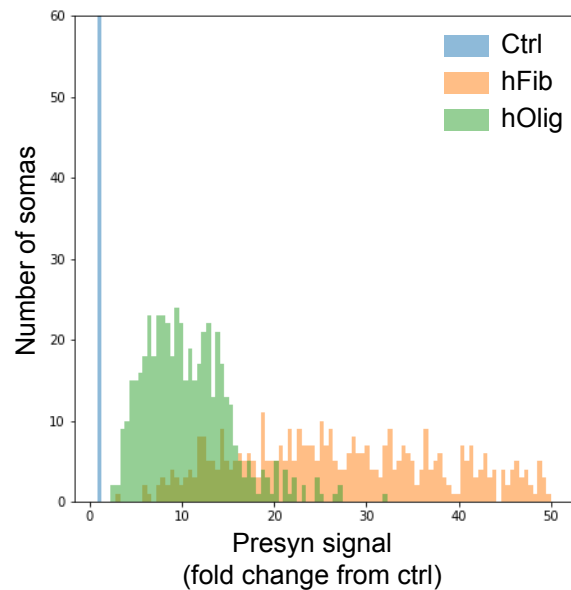**c.**

DIV14

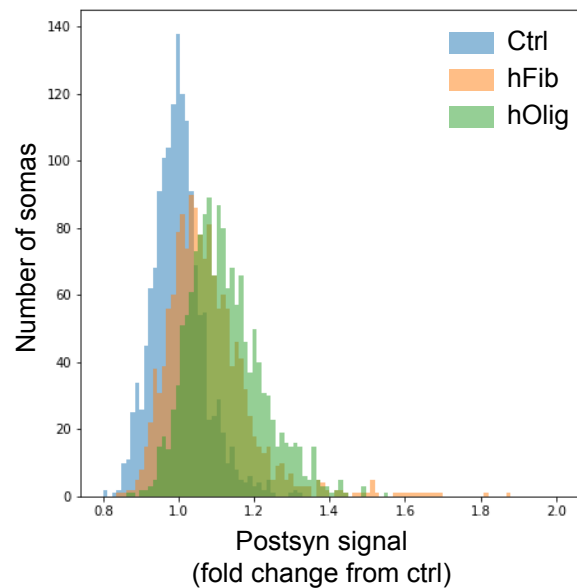**d.**

DIV21

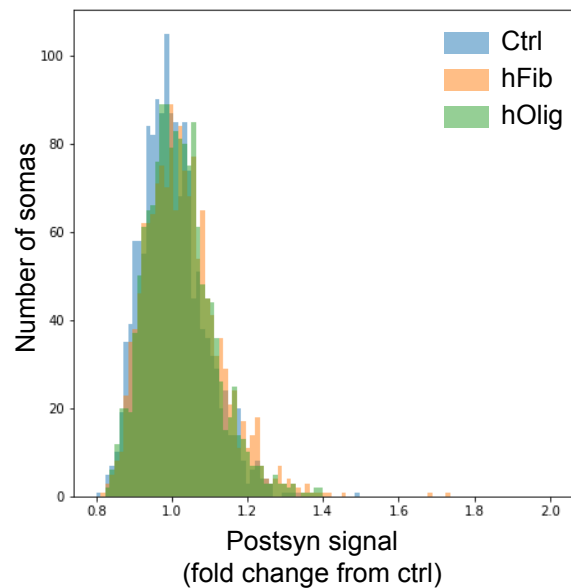

Supplement: Supplementary file 5 — Supplementary Figure 5: Distribution of hFib and hOlig associated fluorescence in presynaptic and postsynaptic neurons. Histograms (100 bins) of the distribution of aggregates associated fluorescence in 500 somas randomly picked from n = 8–21 individual culture devices from N = 2–5 individual experiments. Somas located in the (a, b) presynaptic or (c, d) postsynaptic compartment of Hip>Hip networks treated at (a, c) DIV14 or (b, d) DIV21 with control solution (blue), 500 nM of hFib (orange) or 500 nM hOlig (green). (PDF 521 KB) [file 18_2023_4915_MOESM5_ESM.pdf]

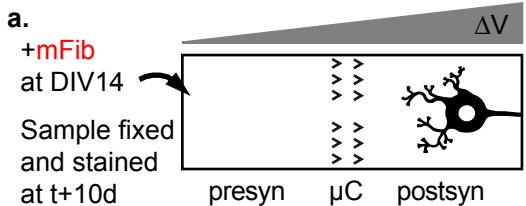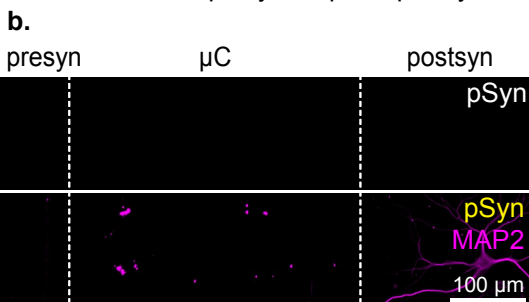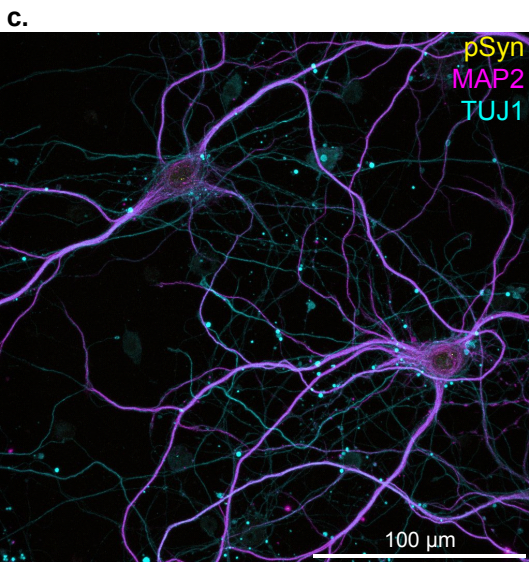

Supplement: Supplementary file 6 — Supplementary Figure 6: Seeding aggregates do not passively diffuse between the culture compartments. a Schematic representation of the experimental design. The presynaptic compartment of Ø>Hip networks was spiked at DIV14 with 500 nM of mFib, and endogenous aSyn aggregation was monitored 10 days later. b Representative epifluorescence field of a Ø>Hip network. c Representative confocal microscopy field of the postsynaptic compartment of a Ø>Hip network. (PDF 2310 KB) [file 18_2023_4915_MOESM6_ESM.pdf]
